# Leading Determinants for Disease-Free Status in Community-Dwelling Middle-Aged Men and Women: A 9-Year Follow-Up Cohort Study

**DOI:** 10.3389/fpubh.2019.00320

**Published:** 2019-11-08

**Authors:** Xianwen Shang, Wei Wang, Stuart Keel, Jinrong Wu, Mingguang He, Lei Zhang

**Affiliations:** ^1^Department of Surgery, Centre for Eye Research Australia, Ophthalmology, University of Melbourne, Melbourne, VIC, Australia; ^2^School of Behavioural and Health Sciences, Australian Catholic University, Melbourne, VIC, Australia; ^3^Department of Medicine-Royal Melbourne Hospital, Faculty of Medicine, Dentistry and Health Sciences, University of Melbourne, Parkville, Melbourne, VIC, Australia; ^4^State Key Laboratory of Ophthalmology, Zhongshan Ophthalmic Center, Sun Yat-sen University, Guangzhou, China; ^5^Melbourne Sexual Health Centre, Alfred Health, Melbourne, VIC, Australia; ^6^Faculty of Medicine, Central Clinical School, Monash University, Melbourne, VIC, Australia; ^7^Department of Epidemiology and Biostatistics, College of Public Health, Zhengzhou University, Zhengzhou, China; ^8^China-Australia Joint Research Centre for Infectious Diseases, School of Public Health, Xi'an Jiaotong University Health Science Center, Xi'an, China

**Keywords:** disease-free status, leading predictors, healthy modifiable factors, family history of chronic disease, socioeconomic status, psychological factors

## Abstract

**Background:** Identifying leading determinants for disease-free status may provide evidence for action priorities, which is imperative for public health with an expanding aged population worldwide. This study aimed to identify leading determinants, especially modifiable factors for disease-free status using machine learning methods.

**Methods:** We included 52,036 participants aged 45–64 years from the 45 and Up Study who were free of 13 predefined chronic conditions at baseline (2006–2009). Disease-free status was defined as participants aging from 45–64 years at baseline to 55–75 years at the end of the follow-up (December 31, 2016) without developing any of the 13 chronic conditions. We used machine learning methods to evaluate the importance of 40 potential predictors and analyzed the association between the number of leading modifiable healthy factors and disease-free status.

**Results:** Disease-free status was found in about half of both men and women during a mean 9-year follow-up. The five most common leading predictors were body mass index (6.4–9.5% of total variance), self-rated health (5.2–8.2%), self-rated quality of life (4.1–6.8%), red meat intake (4.5–6.5%), and chicken intake (4.5–5.9%) in both genders. Modifiable behavioral factors including body mass index, diets, smoking, alcohol consumption, and physical activity, contributed to 37.2–40.3% of total variance. Participants having six or more modifiable health factors were 1.63–8.76 times more likely to remain disease-free status and had 0.60–2.49 more disease-free years (out of 9-year follow-up) than those having two or fewer. Non-behavioral factors including low levels of education and income and high relative socioeconomic disadvantage, were leading risk factors for disease-free status.

**Conclusions:** Body mass index, diets, smoking, alcohol consumption, and physical activity are key factors for disease-free status promotion. Individuals with low socioeconomic status are more in need of care.

## Introduction

The global population is aging, and it is estimated that 16% of the total population will be 65 years or older by 2050 (1). In Australia, 15% of the population were aged ≥65 years in 2014, and the percentage is expected to increase to 23% by 2050 (2, 3). Physiological degeneration with aging is associated with numerous complications, including cardiometabolic disorders, cancer, mental disorders, dementia, Parkinson's disease, musculoskeletal disorders, and asthma (4, 5). These conditions account for a predominant proportion of global mortality with cardiovascular disease and cancer as the first two leading contributors (6). The promotion of disease-free status is an important public health priority, as the prevention of these chronic conditions would notably improve individuals' quality of life and significantly reduce health care costs (7–9).

In 2015, the first world report on healthy aging was released by the World Health Organization (10), and an increasing number of studies have investigated the risk factors for healthy aging (11). Previous studies have linked socioeconomic status and lifestyle, behavioral, psychological, and biological factors to healthy aging (12–15), however; these studies are limited by their cross-sectional design and/or small sample sizes. Although healthy aging is not only the absence of disease (10, 16, 17), disease-free status is the fundamental of healthy aging and is defined by diagnosis of diseases rather than self-rated health with more measurement bias. The importance of determinants in rank on disease-free status is less known (12, 14), thus determining the leading modifiable and non-modifiable predictors based on big data using prediction models especially machine learning considering its advantage in prediction performance is imperative for prioritizing public health actions (18). Middle age represents an important period for chronic disease prevention, therefore identifying the leading determinants for disease-free status during this period is essential (16).

We aimed to prospectively examine the association of lifestyle behaviors, family history of chronic disease, socioeconomic status, psychological and geographic factors with disease-free status and evaluated the importance of 40 potential predictors using machine learning methods based on a large cohort study and claims databases. We also aimed to analyze whether clustering selected leading modifiable factors were associated with disease-free status in men and women.

## Materials and Methods

### Participants

The 45 and Up Study is a prospective study of 266,896 participants aged 45 years and over from New South Wales (19). Participants were randomly sampled from the general population through the Department of Human Services (formerly Medicare Australia) enrolment database and an 18% response rate was achieved, corresponding to 11% of the entire New South Wales population in the target age group (20). Baseline data including lifestyle behaviors, medical history, family history of chronic disease, socioeconomic status, and geographic factors were collected between 2006 and 2009. These data were linked to the Medicare Benefits Schedule and Pharmaceutical Benefits Scheme data (July 1, 2004–December 31, 2016) by the Sax Institute using a unique identifier provided by the Department of Human Services. The 45 and Up study has ethical approval from the UNSW Human Research Ethics Committee. The study protocol was approved by the Royal Victorian Eye and Ear Hospital Human Research Ethics Committee. Participants provided consent to follow-up and link their data to routine health datasets.

**Figure 1:**
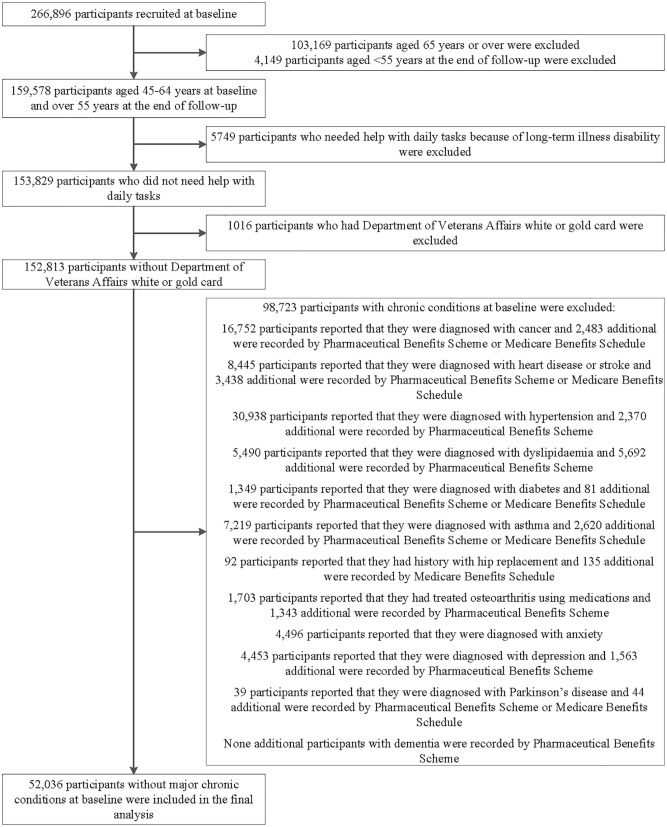
Flowchart of participant selection for the analysis in this study. The main prospective analyses included 52,036 participants aged 45–64 years who were free of major chronic conditions at baseline. We also conducted cross-sectional analysis of the association of individual predictive variable with “disease-free” status and evaluation the importance of variables in 152,813 participants.

This analysis excluded participants with any of the 13 chronic conditions at baseline, including cancer (excluded non-melanoma skin cancer), heart disease, stroke, hypertension, dyslipidemia, diabetes, asthma, depression, anxiety, dementia, Parkinson's disease, hip replacement, and osteoarthritis based on self-reported history of previous diagnosis, Medicare Benefits Schedule, or Pharmaceutical Benefits Scheme claims; those with Department of Veterans' Affairs cards; or those aged 65 years or over; those who needed help with daily tasks because of long-term illness/disability at baseline (Figure 1).

### Independent Variables

Baseline data were collected using a self-administrative questionnaire, which is available at http://www.saxinstitute.org.au/our-work/45-up-study/questionnaires/.

Demographic information including age, gender, ethnicity, income, education, marital status, working status, number of children, and health insurance was assessed. Lifestyle behaviors including dietary intake, smoking, alcohol consumption, physical activity, sleep and sitting time were self-reported based on a questionnaire. Body mass index (BMI) was calculated as weight in kilograms divided by the square of height in meters based on self-reported assessments.

Psychological distress, social interaction, quality of life, and overall health were individually measured using specific indices. Socioeconomic status was assessed using the Index of Relative Socio-economic Disadvantage (21), while geographic remoteness was measured using the Accessibility Remoteness Index of Australia (22). Family history of heart disease, stroke, hypertension, cancer, diabetes, Alzheimer's disease, Parkinson's disease, depression, arthritis, and hip fracture was self-reported. The classification of each independent variable is detailed in Supplementary Methods.

### Outcome Variables

Disease-free status was defined as participants aging from 45–64 years at baseline to 55–75 years at the end of the follow-up without developing any of the 13 chronic conditions. The incidence of the 13 chronic conditions during follow-up was determined by medications and medical services claimed by study participants via the Pharmaceutical Benefits Scheme or Medicare Benefits Schedule (Table S1).

### Statistical Analysis

Descriptive data were summarized as frequency and percentage according to age and gender. We used the Chi-square test to examine whether the incidence of chronic conditions differed by gender and age and Benjamin-Hochberg procedure was used to control the false discovery rate at level 5% for multiple comparisons (23).

The association of potential predictors with disease-free status was assessed using Poisson regression models with robust variance. The multivariable analysis adjusted for age, follow-up period, country of birth, income, education, BMI, psychological distress, smoking, passive smoking, alcohol consumption, physical activity, sleep time, breakfast cereal intake, chicken intake, red meat intake, vegetable intake, fruits intake, health insurance, and social interaction.

We used four established machine learning models including logistic regression, random forest, gradient boost machine, and deep learning to analyze the importance of potential predictors for disease-free status and compared the accuracy of these models (details in Supplementary Methods and Table S2). Twenty leading predictors and 10 leading modifiable factors were obtained according to their contribution derived from machine learning. Poisson regression models with robust variance were then used to analyze the association of clustering 10 leading modifiable healthy factors with disease-free status. A general linear mixed model was used to evaluate the multivariable-adjusted mean difference of disease-free years between participants with a different number of healthy factors. Missing data on each variable examined are listed in Table 1, and those missing values are assigned as a single category.

**Table 1:** Baseline characteristics by gender and age.

	**Men**			**Women**		
	**45–54 years**	**55–64 years**	***P*-value**	**45–54 years**	**55–64 years**	***P*-value**
Country of birth			<0.0001			0.0009
Australia	9,320 (75.2%)	7,278 (71.0%)		13,077 (74.9%)	8,718 (73.1%)	
Others	3,020 (24.4%)	2,912 (28.4%)		4,324 (24.8%)	3,153 (26.4%)	
Missing	54 (0.4%)	63 (0.6%)		67 (0.4%)	50 (0.4%)	
Income			<0.0001			<0.0001
<20,000 AUD	457 (3.7%)	637 (6.2%)		956 (5.5%)	1,102 (9.2%)	
20,000–39,999 AUD	1,039 (8.4%)	1,445 (14.1%)		2,180 (12.5%)	2,043 (17.1%)	
40,000–69,999 AUD	2,822 (22.8%)	2,666 (26.0%)		3,830 (21.9%)	2,673 (22.4%)	
≥70,000 AUD	6,683 (53.9%)	4,080 (39.8%)		7,217 (41.3%)	3,009 (25.2%)	
Missing	1,393 (11.2%)	1,425 (13.9%)		3,285 (18.8%)	3,094 (26.0%)	
Education			<0.0001			<0.0001
<10 years	518 (4.2%)	688 (6.7%)		859 (4.9%)	825 (6.9%)	
High school/TAFE	7,332 (59.2%)	6,186 (60.3%)		10,501 (60.1%)	7,697 (64.6%)	
University or higher	4,436 (35.8%)	3,252 (31.7%)		5,980 (34.2%)	3,285 (27.6%)	
Missing	108 (0.9%)	127 (1.2%)		128 (0.7%)	114 (1.0%)	
Insurance			0.0033			0.5830
Private with extras	6,796 (54.8%)	5,702 (55.6%)		10,012 (57.3%)	6,630 (55.6%)	
Private no extras	1,792 (14.5%)	1,621 (15.8%)		2,291 (13.1%)	1,781 (14.9%)	
Health care concession	594 (4.8%)	669 (6.5%)		975 (5.6%)	1,285 (10.8%)	
None of the above	3,040 (24.5%)	2,107 (20.6%)		3,963 (22.7%)	2,074 (17.4%)	
Missing	172 (1.4%)	154 (1.5%)		227 (1.3%)	151 (1.3%)	
Residential rurality^a^			0.0073			<0.0001
Major cities	6,877 (55.5%)	5,237 (51.1%)		9,335 (53.4%)	5,993 (50.3%)	
Inner regional	3,961 (32.0%)	3,546 (34.6%)		5,956 (34.1%)	4,305 (36.1%)	
Outer regional	1,152 (9.3%)	1,117 (10.9%)		1,639 (9.4%)	1,262 (10.6%)	
Remote	120 (1.0%)	99 (1.0%)		187 (1.1%)	112 (0.9%)	
Missing	284 (2.3%)	254 (2.5%)		351 (2.0%)	249 (2.1%)	
Relative socioeconomic disadvantage^b^			<0.0001			0.0259
1st quintile	1,849 (14.9%)	1,596 (15.6%)		2,577 (14.8%)	1,920 (16.1%)	
2nd quintile	2,225 (18.0%)	1,945 (19.0%)		3,282 (18.8%)	2,272 (19.1%)	
3rd quintile	2,254 (18.2%)	1,879 (18.3%)		3,395 (19.4%)	2,250 (18.9%)	
4th quintile	2,331 (18.8%)	1,884 (18.4%)		3,182 (18.2%)	2,067 (17.3%)	
5th quintile	3,370 (27.2%)	2,617 (25.5%)		4,567 (26.1%)	3,068 (25.7%)	
Missing	365 (2.9%)	332 (3.2%)		465 (2.7%)	344 (2.9%)	
Marital status			0.4555			<0.0001
Married/partner	10,510 (84.8%)	8,731 (85.2%)		14,488 (82.9%)	9,484 (79.6%)	
Single/widowed/divorced	1,884 (15.2%)	1,522 (14.8%)		2,980 (17.1%)	2,437 (20.4%)	
Working status			<0.0001			<0.0001
Working/retired	11,506 (92.8%)	9,143 (89.2%)		13,450 (77.0%)	8,751 (73.4%)	
•Home duty/unpaid work/unemployed/disabled or sick	666 (5.4%)	890 (8.7%)		3,155 (18.1%)	2,712 (22.7%)	
Others	202 (1.6%)	194 (1.9%)		832 (4.8%)	420 (3.5%)	
Missing	20 (0.2%)	26 (0.3%)		31 (0.2%)	38 (0.3%)	
Number of children			<0.0001			<0.0001
None	1,815 (14.6%)	1,166 (11.4%)		2,297 (13.1%)	1,253 (10.5%)	
One or more	10,495 (84.7%)	8,991 (87.7%)		15,116 (86.5%)	10,616 (89.1%)	
Missing	84 (0.7%)	96 (0.9%)		55 (0.3%)	52 (0.4%)	
Family history of cancer			0.0006			<0.0001
No	7,209 (58.2%)	5,732 (55.9%)		9,924 (56.8%)	6,385 (53.6%)	
Yes	5,185 (41.8%)	4,521 (44.1%)		7,544 (43.2%)	5,536 (46.4%)	
Family history of diabetes			<0.0001			<0.0001
No	9,725 (78.5%)	8,401 (81.9%)		13,177 (75.4%)	9,379 (78.7%)	
Yes	2,669 (21.5%)	1,852 (18.1%)		4,291 (24.6%)	2,542 (21.3%)	
Family history of heart disease			<0.0001			<0.0001
No	8,356 (67.4%)	6,540 (63.8%)		10,788 (61.8%)	6,658 (55.9%)	
Yes	4,038 (32.6%)	3,713 (36.2%)		6,680 (38.2%)	5,263 (44.1%)	
Family history of hypertension			<0.0001			<0.0001
No	7,010 (56.6%)	6,468 (63.1%)		7,765 (44.5%)	6,110 (51.3%)	
Yes	5,384 (43.4%)	3,785 (36.9%)		9,703 (55.5%)	5,811 (48.7%)	
Family history of hip fracture			<0.0001			<0.0001
No	11,609 (93.7%)	9,314 (90.8%)		16,267 (93.1%)	10,536 (88.4%)	
Yes	785 (6.3%)	939 (9.2%)		1,201 (6.9%)	1,385 (11.6%)	
Family history of Parkinson's disease			<0.0001			<0.0001
No	11,931 (96.3%)	9,761 (95.2%)		16,737 (95.8%)	11,292 (94.7%)	
Yes	463 (3.7%)	492 (4.8%)		731 (4.2%)	629 (5.3%)	
Family history of stroke			<0.0001			<0.0001
No	10,202 (82.3%)	7,752 (75.6%)		14,049 (80.4%)	8,766 (73.5%)	
Yes	2,192 (17.7%)	2,501 (24.4%)		3,419 (19.6%)	3,155 (26.5%)	
Family history of dementia			<0.0001			<0.0001
No	11,090 (89.5%)	8,381 (81.7%)		15,430 (88.3%)	9,487 (79.6%)	
Yes	1,304 (10.5%)	1,872 (18.3%)		2,038 (11.7%)	2,434 (20.4%)	
Body mass index^c^			0.0054			0.0014
15–18·4 kg/m^2^	64 (0.5%)	58 (0.6%)		294 (1.7%)	190 (1.6%)	
18·5–24·9 kg/m^2^	4,122 (33.3%)	3,582 (34.9%)		9,011 (51.6%)	5,860 (49.2%)	
25–29·9 kg/m^2^	5,698 (46.0%)	4,642 (45.3%)		4,667 (26.7%)	3,490 (29.3%)	
≥30 kg/m^2^	1,937 (15.6%)	1,483 (14.5%)		2,417 (13.8%)	1,635 (13.7%)	
Missing	573 (4.6%)	488 (4.8%)		1,079 (6.2%)	746 (6.3%)	
Smoking			0.0073			<0.0001
Never	7,259 (58.6%)	5,685 (55.4%)		10,988 (62.9%)	8,132 (68.2%)	
Former	3,802 (30.7%)	3,703 (36.1%)		5,043 (28.9%)	3,096 (26.0%)	
Current	1,330 (10.7%)	862 (8.4%)		1,433 (8.2%)	690 (5.8%)	
Missing	3 (0.0%)	3 (0.0%)		4 (0.0%)	3 (0.0%)	
Passive smoking			0.0011			0.8708
Yes	7,211 (58.2%)	6,256 (61.0%)		11,477 (65.7%)	7,953 (66.7%)	
No	4,319 (34.8%)	3,172 (30.9%)		4,794 (27.4%)	2,856 (24.0%)	
Missing	864 (7.0%)	825 (8.0%)		1,197 (6.9%)	1,112 (9.3%)	
Alcohol consumption			0.0162			0.7865
None	2,415 (19.5%)	1,897 (18.5%)		5,425 (31.1%)	3,776 (31.7%)	
1–4 sessions/week	2,575 (20.8%)	2,022 (19.7%)		4,617 (26.4%)	2,957 (24.8%)	
5–7 sessions/week	1,810 (14.6%)	1,525 (14.9%)		2,809 (16.1%)	2,058 (17.3%)	
7–14 sessions/week	2,571 (20.7%)	2,287 (22.3%)		3,199 (18.3%)	2,255 (18.9%)	
≥15 sessions/week	2,921 (23.6%)	2,445 (23.8%)		1,247 (7.1%)	751 (6.3%)	
Missing	102 (0.8%)	77 (0.8%)		171 (1.0%)	124 (1.0%)	
Physical activity			<0.0001			<0.0001
0–4 sessions/week	2,207 (17.8%)	1,609 (15.7%)		2,767 (15.8%)	1,524 (12.8%)	
5–9 sessions/week	3,420 (27.6%)	2,667 (26.0%)		5,288 (30.3%)	3,589 (30.1%)	
10–14 sessions/week	2,611 (21.1%)	2,330 (22.7%)		4,345 (24.9%)	3,062 (25.7%)	
≥15 sessions/week	3,884 (31.3%)	3373 (32.9%)		4,709 (27.0%)	3,474 (29.1%)	
Missing	272 (2.2%)	274 (2.7%)		359 (2.1%)	272 (2.3%)	
Sleep time			<0.0001			0.4723
<7 h	2,011 (16.2%)	1,517 (14.8%)		2,241 (12.8%)	1,607 (13.5%)	
7–9 h	9,937 (80.2%)	8,245 (80.4%)		14,487 (82.9%)	9,769 (81.9%)	
>9 h	208 (1.7%)	301 (2.9%)		458 (2.6%)	320 (2.7%)	
Missing	238 (1.9%)	190 (1.9%)		282 (1.6%)	225 (1.9%)	
Sitting time			<0.0001			<0.0001
8 h	7,717 (62.3%)	7,006 (68.3%)		12,144 (69.5%)	8,841 (74.2%)	
≥8 h	4,185 (33.8%)	2,798 (27.3%)		4,447 (25.5%)	2,381 (20.0%)	
Missing	492 (4.0%)	449 (4.4%)		877 (5.0%)	699 (5.9%)	
Outdoor physical activity			<0.0001			<0.0001
≤ 12 h/week	2,491 (20.1%)	1,560 (15.2%)		5,958 (34.1%)	3,363 (28.2%)	
12·9–20 h/week	3,171 (25.6%)	2,321 (22.6%)		5,492 (31.4%)	3,595 (30.2%)	
20·1–30 h/week	2,465 (19.9%)	2,317 (22.6%)		3,180 (18.2%)	2,654 (22.3%)	
>30 h/week	4,153 (33.5%)	3,951 (38.5%)		2,658 (15.2%)	2,156 (18.1%)	
Missing	114 (0.9%)	104 (1.0%)		180 (1.0%)	153 (1.3%)	
Breakfast cereal intake			0.1734			0.0003
Non-high fiber	2,022 (16.3%)	1,505 (14.7%)		1,979 (11.3%)	1,345 (11.3%)	
High fiber	7,877 (63.6%)	6,735 (65.7%)		11,837 (67.8%)	8,363 (70.2%)	
Missing	2,495 (20.1%)	2,013 (19.6%)		3,652 (20.9%)	2,213 (18.6%)	
Milk intake			<0.0001			<0.0001
None	579 (4.7%)	562 (5.5%)		764 (4.4%)	662 (5.6%)	
Skimmed fat/reduced fat/soy milk	5,814 (46.9%)	5,093 (49.7%)		11,118 (63.6%)	7,985 (67.0%)	
Whole milk	5,753 (46.4%)	4,341 (42.3%)		5,128 (29.4%)	2,915 (24.5%)	
Missing	248 (2.0%)	257 (2.5%)		458 (2.6%)	359 (3.0%)	
Chicken intake			<0.0001			<0.0001
None	377 (3.0%)	457 (4.5%)		662 (3.8%)	561 (4.7%)	
1 serving per week	1,883 (15.2%)	2,102 (20.5%)		2,477 (14.2%)	2,223 (18.6%)	
2 servings per week	3,457 (27.9%)	2,939 (28.7%)		4,895 (28.0%)	3,425 (28.7%)	
3 or more servings per week	3,955 (31.9%)	2,704 (26.4%)		5,594 (32.0%)	3,286 (27.6%)	
Missing	2,722 (22.0%)	2,051 (20.0%)		3,840 (22.0%)	2,426 (20.4%)	
Fish intake			<0.0001			<0.0001
None	1,231 (9.9%)	753 (7.3%)		1,667 (9.5%)	797 (6.7%)	
1 serving per week	5,619 (45.3%)	4,531 (44.2%)		7,426 (42.5%)	4,463 (37.4%)	
2 or more servings per week	4,896 (39.5%)	4,388 (42.8%)		7,404 (42.4%)	6,052 (50.8%)	
Missing	648 (5.2%)	581 (5.7%)		971 (5.6%)	609 (5.1%)	
Red meat intake			0.2114			0.0348
0 or 1 serving per week	1,317 (10.6%)	1,103 (10.8%)		2,549 (14.6%)	1,683 (14.1%)	
2 servings per week	2,144 (17.3%)	1,651 (16.1%)		3,151 (18.0%)	1,906 (16.0%)	
3 or 4 servings per week	3,920 (31.6%)	3,419 (33.3%)		5,887 (33.7%)	4,169 (35.0%)	
5 or more servings per week	2,332 (18.8%)	2,139 (20.9%)		2,065 (11.8%)	1,779 (14.9%)	
Missing	2,681 (21.6%)	1,941 (18.9%)		3,816 (21.8%)	2,384 (20.0%)	
Processed meat intake			<0.0001			0.1015
0 or 1 serving per week	1,899 (15.3%)	1,769 (17.3%)		4,851 (27.8%)	3,713 (31.1%)	
2 or more servings per week	9,451 (76.3%)	7,276 (71.0%)		10,549 (60.4%)	6,468 (54.3%)	
Missing	1,044 (8.4%)	1,208 (11.8%)		2,068 (11.8%)	1,740 (14.6%)	
Vegetables intake			<0.0001			<0.0001
0 or 1 serving per day	2,070 (16.7%)	1,617 (15.8%)		1,110 (6.4%)	577 (4.8%)	
2 servings per day	4,252 (34.3%)	3,235 (31.6%)		4,119 (23.6%)	2,327 (19.5%)	
3 servings per day	2,031 (16.4%)	1,624 (15.8%)		3,137 (18.0%)	1,858 (15.6%)	
4 servings per day	1,478 (11.9%)	1,277 (12.5%)		3,126 (17.9%)	2,226 (18.7%)	
5 or more servings per day	2,292 (18.5%)	2,227 (21.7%)		5,487 (31.4%)	4,592 (38.5%)	
Missing	271 (2.2%)	273 (2.7%)		489 (2.8%)	341 (2.9%)	
Fruits intake			<0.0001			<0.0001
None	1,309 (10.6%)	891 (8.7%)		1,006 (5.8%)	459 (3.9%)	
1 serving per day	4,612 (37.2%)	3,773 (36.8%)		5,545 (31.7%)	3,011 (25.3%)	
2 servings per day	3,377 (27.2%)	2,837 (27.7%)		6,032 (34.5%)	4,378 (36.7%)	
3 or more servings per day	2,452 (19.8%)	2,148 (20.9%)		4,104 (23.5%)	3,538 (29.7%)	
Missing	644 (5.2%)	604 (5.9%)		781 (4.5%)	535 (4.5%)	
Psychological distress^d^			<0.0001			<0.0001
Low	5,345 (43.1%)	5,393 (52.6%)		6,710 (38.4%)	5,880 (49.3%)	
Mild	4,722 (38.1%)	3,386 (33.0%)		6,982 (40.0%)	3,929 (33.0%)	
Moderate	1,716 (13.8%)	933 (9.1%)		2,589 (14.8%)	1,163 (9.8%)	
High	408 (3.3%)	208 (2.0%)		711 (4.1%)	281 (2.4%)	
Missing	203 (1.6%)	333 (3.2%)		476 (2.7%)	668 (5.6%)	
Social interaction^e^			<0.0001			<0.0001
Low	2,521 (20.3%)	1,938 (18.9%)		2,752 (15.8%)	1,453 (12.2%)	
Moderate	7,341 (59.2%)	5,837 (56.9%)		10,774 (61.7%)	6,795 (57.0%)	
High	1,815 (14.6%)	1,804 (17.6%)		2,850 (16.3%)	2,836 (23.8%)	
Missing	717 (5.8%)	674 (6.6%)		1,092 (6.3%)	837 (7.0%)	
Self-rated health status			<0.0001			<0.0001
Excellent	3,013 (24.3%)	2,697 (26.3%)		5,463 (31.3%)	3,885 (32.6%)	
Very good	5,220 (42.1%)	4,599 (44.9%)		7,345 (42.0%)	5,365 (45.0%)	
Good	3,322 (26.8%)	2,331 (22.7%)		3,599 (20.6%)	1,999 (16.8%)	
Fair/Poor	540 (4.4%)	329 (3.2%)		489 (2.8%)	246 (2.1%)	
Missing	299 (2.4%)	297 (2.9%)		572 (3.3%)	426 (3.6%)	
Self-rated quality of life			<0.0001			<0.0001
Excellent	2,979 (24.0%)	2,658 (25.9%)		5,372 (30.8%)	3,815 (32.0%)	
Very good	5,158 (41.6%)	4,533 (44.2%)		7,258 (41.6%)	5,274 (44.2%)	
Good	3,273 (26.4%)	2,281 (22.2%)		3,551 (20.3%)	1,946 (16.3%)	
Fair/Poor	530 (4.3%)	324 (3.2%)		483 (2.8%)	238 (2.0%)	
Missing	454 (3.7%)	457 (4.5%)		804 (4.6%)	648 (5.4%)	

a *Residential rurality was categorized as four groups including major cities, inner regional area, outer regional area, and remoteness using the Accessibility Remoteness Index of Australia*.

b *Relative socioeconomic disadvantage was divided into quintiles, with the lowest quintile representing the greatest socio-economic disadvantage*.

c *Body mass index was calculated as weight in kilograms divided by the square of height in meters*.

d *Psychological distress measured by the Kessler-10 scale provides a global measure of anxiety and depressive symptoms experienced in the preceding month, with the following categories: low, mild, moderate, and high psychological distress*.

e *Social interaction was categorized as low, mild, moderate, and high levels using the Duke Social Support Scale*.

Sensitivity analysis was conducted to examine the cross-sectional associations of potential predictors with “disease-free” and leading predictors using the baseline data with 152,813 participants aged 45–64 years, where disease-free was defined as being free of the 13 chronic conditions at baseline.

We realized these machine learning modeling exercises using the statistical software R 3.4.1. Other analyses were performed using SAS version 9.4 (SAS Institute Inc.), and all *P*-values were two-sided.

## Results

### Participant Characteristics

As shown in Table 1, 52,036 participants aged 45–64 years (56.9% female) with a mean follow-up of 8.9 ± 0.9 (range: 7.0, 11.5) years were included in the analysis. Individuals aged 45–54 years had higher income, education, the prevalence of overweight/obesity, smoking prevalence and consumed less vegetable, fruit, and fish and more chicken compared with those aged 55–64 years in both men and women (all *P* < 0.0001). Younger individuals were less likely to report an excellent self-rated quality of life or overall health compared to their older counterparts (all *P* < 0.0001).

### Disease-Free Status by Age and Gender

**Figure 2:**
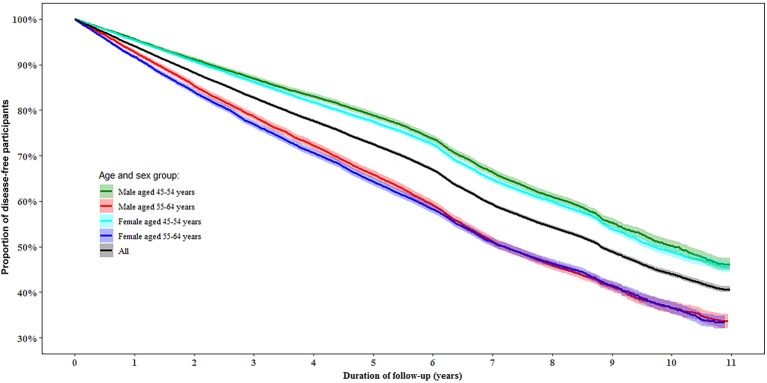
Proportion of disease-free status by age and gender during follow-up. Disease-free status was defined as participants aging from 45–64 years at baseline to 55–75 years at the end of the follow-up without developing any of the 13 chronic conditions.

During follow-up, 50.0% of the participants were determined as disease-free status with a similar proportion in women (49.8%) and men (50.1%). In the multivariable-adjusted model, men aged 55–64 years had a 45% (95% confidence interval [CI]: 41, 48%) lower likelihood of disease-free status compared to those aged 45–54 years (*P* < 0.0001). While women aged 55–64 years had a 38% (95% CI: 35, 41%) lower likelihood of disease-free status than those aged 45–54 years (*P* < 0.0001, Figure 2).

### Incidence of Individual Chronic Conditions by Age and Gender

**Figure 3:**
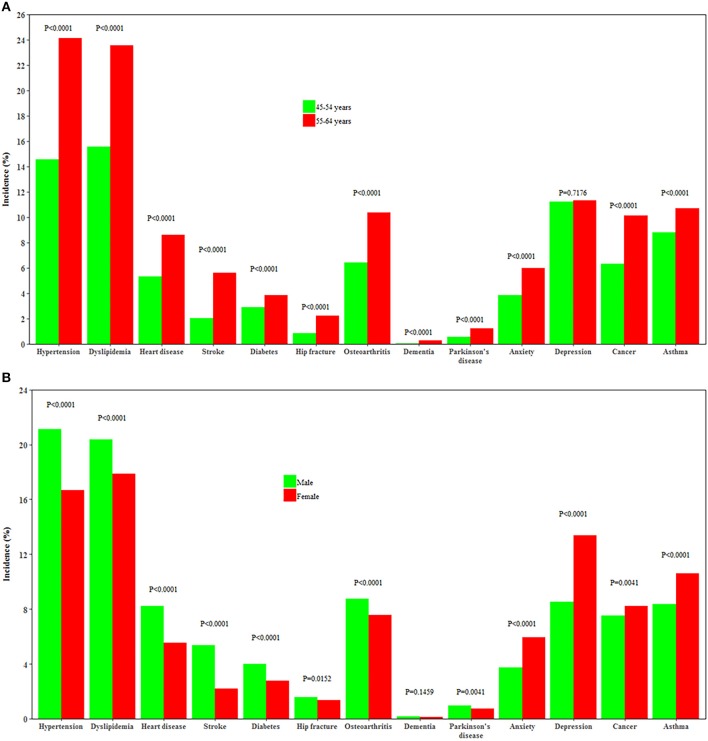
Incidence of 13 chronic conditions by age and gender. **(A,B)** Show the incidence of 13 chronic conditions by age and by gender, respectively.

Individuals aged 55–64 years had a higher incidence of all chronic conditions except depression than those aged 45–54 years (all *P* < 0.0001). Men had a higher incidence of heart disease, stroke, hypertension, dyslipidemia, diabetes, Parkinson's disease, osteoarthritis, and hip replacement, while women had a higher incidence of depression, anxiety, cancer, and asthma (all *P* < 0.0001, Figure 3).

### Relative Risk for Disease-Free Status Associated With Potential Predictors

In the multivariable analysis, a smaller proportion of disease-free status was observed in participants with overweight [Relative risk (RR) 0.72 (95% CI: 0.69, 0.75)] or obesity [0.48 (95% CI: 0.46, 0.51)], older age [0.88 (95% CI: 0.86, 0.89)] for each year increase, smoking [0.67 (95% CI: 0.63, 0.72)], passive smoking [0.94 (95% CI: 0.90, 0.98)], excessive alcohol consumption [0.93 (95% CI: 0.87, 0.99)], diets high in chicken [0.77 (95% CI: 0.70, 0.85)], moderate [0.81 (95% CI: 0.77, 0.86)] or high psychological distress [0.66 (95% CI: 0.59, 0.73)], poor/fair self-rated health [0.56 (95% CI: 0.50, 0.63)], or quality of life [0.56 (95% CI: 0.50, 0.63)], and family history of heart disease [0.80 (95% CI: 0.77, 0.83)], stroke [0.86 (95% CI: 0.82, 0.89)], or hypertension [0.86 (95% CI: 0.82, 0.89)] (all *P* < 0.05). High level of physical activity [RR: 1.13 (95% CI: 1.07, 1.20)], sleep time between 7 and 9 h [1.07 (95% CI: 1.02, 1.13)], high education [1.57 (95% CI: 1.44, 1.71)] were associated with a higher likelihood of disease-free status (all *P* < 0.05, Table 2).

**Table 2:** Relative risks for disease-free status associated with potential predictors.

	**Disease-free participants^**a**^**	**Person-years**	**Proportion of disease-free status**	**Univariate analysis**		**Multivariate analysis**^**b**^	
				**Relative risk (95% CI)**	***P*-value**	**Relative risk (95% CI)**	***P*-value**
Age (per 2 years)				0.89 (0.88–0.89)	<0.0001	0.88 (0.86–0.89)	<0.0001
Gender					0.6122		<0.0001
Male	11,287	152,536	49.8	1.00 (1.00–1.00)		1.00 (1.00–1.00)	
Female	14,713	196,553	50.1	1.01 (0.97–1.04)		0.90 (0.87–0.94)	
Country of birth					0.0019		0.6067
Australia	19,047	257,782	49.6	1.00 (1.00–1.00)		1.00 (1.00–1.00)	
Others	6,861	89,873	51.2	1.06 (1.02–1.11)		1.01 (0.97–1.05)	
Income					<0.0001		0.0318
<20,000 AUD	1,430	19,715	45.4	1.00 (1.00–1.00)		1.00 (1.00–1.00)	
20,000–39,999 AUD	3,166	43,812	47.2	1.08 (0.99–1.17)		0.99 (0.91–1.09)	
40,000–69,999 AUD	6,032	81,105	50.3	1.22 (1.13–1.32)		1.08 (0.99–1.18)	
≥70,000 AUD	11,179	145,042	53.3	1.37 (1.27–1.48)		1.08 (0.99–1.18)	
Education level					<0.0001		<0.0001
< 10 years	1,092	17,247	37.8	1.00 (1.00–1.00)		1.00 (1.00–1.00)	
High school/TAFE	15,327	210,291	48.3	1.54 (1.42–1.67)		1.33 (1.22–1.44)	
University or higher	9,372	118,559	55.3	2.04 (1.88–2.21)		1.57 (1.44–1.71)	
Insurance					<0.0001		<0.0001
Private with extras	14,284	194,774	49.0	1.00 (1.00–1.00)		1.00 (1.00–1.00)	
Private no extras	3,872	51,465	51.7	1.11 (1.06–1.17)		1.14 (1.08–1.20)	
Health care concession	1,512	21,000	42.9	0.78 (0.73–0.84)		1.04 (0.96–1.13)	
None of the above	5,986	77,239	53.5	1.20 (1.15–1.25)		1.33 (1.27–1.39)	
Residential rurality					0.0143		<0.0001
Major cities	13,593	183,581	49.5	1.00 (1.00–1.00)		1.00 (1.00–1.00)	
Inner regional	9,032	120,002	50.8	1.05 (1.01–1.09)		1.11 (1.07–1.16)	
Outer regional	2,539	34,365	49.1	0.98 (0.93–1.04)		1.07 (1.01–1.14)	
Remote	244	3,373	47.1	0.91 (0.76–1.08)		1.04 (0.87–1.25)	
Relative socioeconomic disadvantage					<0.0001		0.1390
1st quintile	3,694	51,583	46.5	1.00 (1.00–1.00)		1.00 (1.00–1.00)	
2nd quintile	4,781	64,786	49.2	1.11 (1.05–1.18)		1.06 (1.00–1.13)	
3rd quintile	4,928	65,968	50.4	1.17 (1.10–1.24)		1.09 (1.02–1.15)	
4th quintile	4,763	63,841	50.3	1.17 (1.10–1.24)		1.05 (0.99–1.12)	
5th quintile	7,068	92,747	51.9	1.24 (1.17–1.31)		1.05 (0.99–1.12)	
Marital status					0.8435		0.0812
Married/Partner	21,600	290,356	50.0	1.00 (1.00–1.00)		1.00 (1.00–1.00)	
Single/Widowed/Divorced	4,400	58,732	49.9	1.00 (0.95–1.04)		1.05 (0.99-1.10)	
Working status					0.0006		0.6842
Working/retired	21,547	289,196	50.3	1.00 (1.00–1.00)		1.00 (1.00–1.00)	
Home duty/unpaid work/unemployed/disabled or sick	3,558	48,098	47.9	0.91 (0.87–0.96)		0.98 (0.93–1.03)	
Others	841	11,055	51.0	1.03 (0.93–1.14)		0.99 (0.89–1.10)	
Number of children					<0.001		0.3592
None	3,445	44,639	52.8	1.00 (1.00–1.00)		1.00 (1.00–1.00)	
One or more	22,438	302,600	49.6	0.88 (0.84–0.93)		0.97 (0.92–1.03)	
Family history of cancer					0.7355		0.4845
No	14,634	196,578	50.0	1.00 (1.00–1.00)		1.00 (1.00–1.00)	
Yes	11,366	152,510	49.9	0.99 (0.96–1.03)		1.01 (0.98–1.05)	
Family history of diabetes					0.0011		0.0327
No	20,481	273,982	50.3	1.00 (1.00–1.00)		1.00 (1.00–1.00)	
Yes	5,519	75,107	48.6	0.93 (0.89–0.97)		0.95 (0.91–1.00)	
Family history of heart disease					<0.0001		<0.0001
No	16,887	221,172	52.2	1.00 (1.00–1.00)		1.00 (1.00–1.00)	
Yes	9,113	127,916	46.3	0.79 (0.76–0.82)		0.80 (0.77–0.83)	
Family history of hypertension					<0.0001		<0.0001
No	13,945	184,345	51.0	1.00 (1.00–1.00)		1.00 (1.00–1.00)	
Yes	12,055	164,743	48.8	0.92 (0.89–0.95)		0.86 (0.83–0.89)	
Family history of stroke					<0.0001		<0.0001
No	20,812	275,908	51.1	1.00 (1.00–1.00)		1.00 (1.00–1.00)	
Yes	5,188	73,180	46.1	0.82 (0.78–0.85)		0.86 (0.82–0.89)	
Body mass index					<0.0001		<0.0001
15–18.4 kg/m^2^	355	4,322	58.6	1.00 (1.00–1.00)		1.00 (1.00–1.00)	
18.5–24.9 kg/m^2^	12,687	158,790	56.2	0.91 (0.77–1.07)		0.83 (0.70–0.98)	
25–29.9 kg/m^2^	8,769	121,625	47.4	0.64 (0.54–0.75)		0.60 (0.51–0.71)	
≥30 kg/m^2^	2,765	45,221	37.0	0.42 (0.35–0.49)		0.40 (0.34–0.48)	
Smoking					<0.0001		<0.0001
Never	16,798	219,507	52.4	1.00 (1.00–1.00)		1.00 (1.00–1.00)	
Former	7,419	102,443	47.4	0.82 (0.79–0.85)		0.87 (0.83–0.90)	
Current	1,778	27,075	41.2	0.64 (0.60–0.68)		0.67 (0.63–0.72)	
Passive smoking					<0.0001		0.0039
No	17,068	222,930	51.9	1.00 (1.00–1.00)		1.00 (1.00–1.00)	
Yes	7,065	100,428	46.7	0.81 (0.78–0.84)		0.94 (0.90–0.98)	
Alcohol consumption					<0.0001		0.0004
None	6,707	89,581	49.6	1.00 (1.00–1.00)		1.00 (1.00–1.00)	
1–4 drinks/week	6,228	83,001	51.2	1.06 (1.01–1.12)		1.05 (1.00–1.11)	
5–7 drinks/week	4,250	55,813	51.8	1.09 (1.03–1.15)		1.06 (1.00–1.12)	
7–14 drinks/week	5,240	69,636	50.8	1.05 (1.00–1.10)		1.04 (0.98–1.10)	
≥15 drinks/week	3,365	47,942	45.7	0.85 (0.81–0.90)		0.93 (0.87–0.99)	
Physical activity					<0.001		0.0006
0–4 sessions/week	3,790	52,768	46.8	1.00 (1.00–1.00)		1.00 (1.00–1.00)	
5–9 sessions/week	7,471	100,829	49.9	1.14 (1.08–1.20)		1.09 (1.03–1.15)	
10–14 sessions/week	6,241	83,237	50.5	1.16 (1.10–1.23)		1.09 (1.03–1.16)	
≥15 sessions/week	7,975	104,841	51.7	1.22 (1.15–1.28)		1.13 (1.07–1.20)	
Outdoor physical activity					<0.0001		0.1062
≤ 12 h/week	6,828	91,051	51.1	1.00 (1.00–1.00)		1.00 (1.00–1.00)	
12.9–20 h/week	7,391	98,357	50.7	0.99 (0.94–1.03)		1.02 (0.97–1.07)	
20.1–30 h/week	5,338	71,581	50.3	0.97 (0.92–1.02)		1.07 (1.01–1.13)	
>30 h/week	6,211	84,713	48.1	0.89 (0.85–0.93)		1.04 (0.99–1.10)	
Sleeping time					<0.0001		0.0088
<7 h/day	3,413	48,564	46.3	1.00 (1.00–1.00)		1.00 (1.00–1.00)	
7–9 h/day	21,562	286,315	50.8	1.20 (1.14–1.26)		1.07 (1.02–1.13)	
>9 h/day	583	8,065	45.3	0.96 (0.85–1.08)		0.97 (0.86–1.10)	
Sitting time					<0.0001		0.0215
<8 h/day	17,682	238,839	49.5	1.00 (1.00–1.00)		1.00 (1.00–1.00)	
≥8 h/day	7,164	93,986	51.9	1.10 (1.06–1.14)		1.05 (1.01–1.10)	
Breakfast cereal intake					<0.0001		0.2649
Non-high fiber	3,303	45,340	48.2	1.00 (1.00–1.00)		1.00 (1.00–1.00)	
High fiber	17,753	235,206	51.0	1.12 (1.06–1.18)		1.03 (0.98–1.09)	
Milk intake					<0.0001		<0.0001
None	1,273	17,092	49.6	1.00 (1.00–1.00)		1.00 (1.00–1.00)	
Skimmed fat/reduced fat/soy milk	14,661	199,717	48.9	0.97 (0.90–1.05)		0.92 (0.85–1.00)	
Whole milk	9,508	124,056	52.4	1.12 (1.03–1.22)		1.10 (1.01–1.20)	
Chicken intake					<0.0001		<0.0001
None	1,229	14,082	59.8	1.00 (1.00–1.00)		1.00 (1.00–1.00)	
1 serving/week	4,560	56,491	52.5	0.74 (0.68–0.82)		0.83 (0.75–0.92)	
2 servings/week	7,696	96,213	52.3	0.74 (0.67–0.81)		0.81 (0.73–0.90)	
3 or more servings/week	7,945	101,030	51.1	0.70 (0.64–0.77)		0.77 (0.70–0.85)	
Fish intake					<0.0001		0.0010
None	2,344	30,379	52.7	1.00 (1.00–1.00)		1.00 (1.00–1.00)	
1 serving/week	11,191	149,262	50.8	0.93 (0.87–0.99)		0.98 (0.91–1.05)	
2 or more servings/week	11,181	151,470	49.2	0.87 (0.81–0.93)		0.91 (0.85–0.98)	
Red meat intake					<0.0001		0.1383
0 or 1 serving/week	3,735	44,462	56.2	1.00 (1.00–1.00)		1.00 (1.00–1.00)	
2 servings/week	4,666	58,289	52.7	0.87 (0.82–0.93)		0.94 (0.88–1.01)	
3 or 4 servings/week	8,929	112,952	51.3	0.82 (0.78–0.87)		0.93 (0.87–0.99)	
5 or more servings/week	4,209	53,444	50.6	0.80 (0.75–0.85)		0.96 (0.89–1.03)	
Vegetables intake					<0.0001		0.0919
0 or 1 serving/day	2,560	35,800	47.6	1.00 (1.00–1.00)		1.00 (1.00–1.00)	
2 servings/day	7,060	94,687	50.7	1.13 (1.06–1.20)		1.06 (0.99–1.13)	
3 servings/day	4,472	58,905	51.7	1.18 (1.10–1.26)		1.05 (0.98–1.13)	
4 servings/day	4,075	54,403	50.3	1.11 (1.04–1.19)		1.00 (0.92–1.07)	
5 or more servings/day	7,261	96,825	49.7	1.09 (1.02–1.16)		1.01 (0.95–1.09)	
Fruits intake					<0.0001		0.0425
None	1,713	23,915	46.7	1.00 (1.00–1.00)		1.00 (1.00–1.00)	
1 serving/day	8,551	114,014	50.5	1.16 (1.08–1.25)		1.05 (0.97–1.13)	
2 servings/day	8,326	111,549	50.1	1.14 (1.06–1.23)		1.00 (0.92–1.08)	
3 or more servings/day	6,335	83,465	51.8	1.22 (1.13–1.32)		1.06 (0.98–1.15)	
Social interaction					0.0016		0.0277
Low	4,398	58,713	50.8	1.00 (1.00–1.00)		1.00 (1.00–1.00)	
Moderate	15,549	207,435	50.6	0.99 (0.95–1.04)		0.97 (0.92–1.02)	
High	4,518	61,526	48.6	0.92 (0.86–0.97)		0.92 (0.87–0.98)	
Psychological distress					<0.0001		<0.0001
Low	11,925	159,193	51.1	1.00 (1.00–1.00)		1.00 (1.00–1.00)	
Mild	9,606	127,305	50.5	0.98 (0.94–1.01)		0.89 (0.86–0.93)	
Moderate	3,097	42,150	48.4	0.90 (0.85–0.95)		0.81 (0.77–0.86)	
High	681	9,950	42.4	0.70 (0.63–0.78)		0.66 (0.59–0.73)	
Self-rated health status					<0.0001		<0.0001
Excellent	8,664	107,084	57.5	1.00 (1.00–1.00)		1.00 (1.00–1.00)	
Very good	11,165	150,953	49.6	0.73 (0.70–0.76)		0.80 (0.76–0.83)	
Good	4,889	71,749	43.5	0.57 (0.54–0.60)		0.67 (0.63–0.71)	
Fair/Poor	592	9,328	36.9	0.43 (0.39–0.48)		0.56 (0.50–0.63)	
Self-rated quality of life					<0.0001		<0.0001
Excellent	8,535	105,451	57.6	1.00 (1.00–1.00)		1.00 (1.00–1.00)	
Very good	11,034	149,060	49.7	0.73 (0.70–0.76)		0.80 (0.77–0.84)	
Good	4,817	70,548	43.6	0.57 (0.54–0.60)		0.67 (0.63–0.71)	
Fair/Poor	580	9,178	36.8	0.43 (0.39–0.48)		0.56 (0.50–0.63)	

a *Disease-free status was defined as participants aging from 45–64 years at baseline to 55–75 years at the end of the follow-up (December 31, 2016) without developing any of the 13 chronic conditions. Poisson regression model with robust variance was used to estimate relative risks (95% confidence intervals)*.

b *Multivariate relative risks were adjusted for age, follow-up period, country of birth, income, education, body mass index, psychological distress, smoking, passive smoking, alcohol consumption, physical activity, sleep time, breakfast cereal intake, chicken intake, red meat intake, vegetables intake, fruits intake, healthy insurance, and social interaction*.

### Importance of Contributors to Disease-Free Status

**Figure 4:**
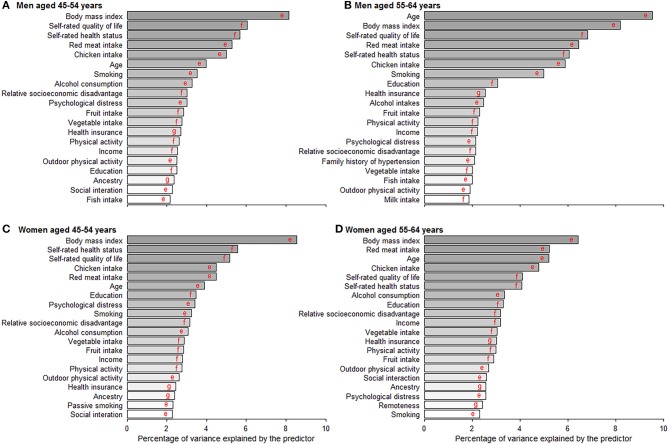
Age- and gender-specific 20 leading predictors for disease-free status derived from random forest. **(A–D)** Show the 20 leading predictors for disease-free status in men aged 45–54 and 55–64 years and women aged 45–54 and 55–64 years. Disease-free status was defined as participants aging from 45–64 years at baseline to 55–75 years at the end of the follow-up without developing any of the 13 chronic conditions. Machine learning methods including random forest, gradient boosting machine, deep learning, and logistic regression were applied to evaluate the importance of predictors and results from random forest with the best prediction performance are shown in this figure. ^e^Variables were inversely associated with disease-free status proportion. ^f^Variables were positively associated with disease-free status proportion. ^g^Variables were non-linearly associated with disease-free status proportion.

Random Forest exhibited a higher prediction performance (as assessed by area under the curve) compared with the other three machine learning models (Table S3). Figure 4 depicts the leading predictors for disease-free status in women and men, stratified by age group, as derived from Random Forest. For both men and women, although in different orders, the six leading predictors for disease-free status were BMI (range: 6.4, 9.5% of total variance), self-rated life quality (4.1, 6.8%), self-rated health (4.1, 6.0%), red meat intake (4.5, 6.5%), chicken intake (4.5, 5.9%), and age (3.9, 9.5%). Age was ranked as the sixth leading predictor (4.0% total variance) for disease-free status in men aged 45–54 years but was the most important predictor (9.5%) at age 55–64 years. Results from other machine learning methods shown in Table S4.

### Clustering Modifiable Healthy Factors and Disease-Free Status Years and Proportion According to Age and Gender

**Figure 5:**
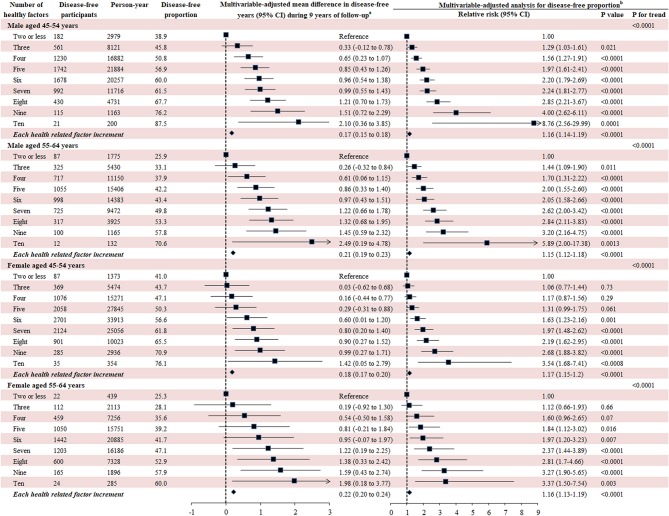
Number of modifiable healthy factors and disease-free years and proportion. Disease-free status was defined as participants aging from 45–64 years at baseline to 55–75 years at the end of the follow-up without developing any of the 13 chronic conditions. The 10 leading modifiable healthy factors included BMI between 18.5 and 24.9 kg/m^2^, fruit intake ≥ 2 servings/day, vegetables intake ≥ 3 servings/day, physical activity ≥ 5 sessions/week, red meat intake ≤ 1 serving/week, chicken intake ≤ 1 serving/week, alcohol consumption between 1 and 4 drinks/week, never smoking, none passive smoking, regular whole milk drinking. ^a^Generalized linear regression model was used to evaluate the mean difference of disease-free years between different participants with number of healthy factors with the same covariates adjusted for in the Poisson regression analysis. ^b^Multivariate analysis was conducted using Poisson regression model with robust variance adjusted for age, follow-up period, country of birth, income, education, psychological distress, remoteness, marital status, healthy insurance, self-rated health, self-rated quality of life smoking, and family history of cardiovascular disease, cancer, diabetes, hypertension, hip fracture, Parkinson's disease, and dementia.

The 10 leading modifiable factors for disease-free status contributed to 37.2–40.3% of the total variance across all subgroups. We defined these 10 modifiable healthy factors as normal weight, high physical activity, moderate alcohol consumption, never smoking, none passive smoking, and diets high in fruit, vegetables, and whole milk and low in red and chicken according to their association with disease-free status. A higher proportion of women (59.5%) displayed six or more healthy factors than men (43.1%), and older participants had more healthy factors than their younger counterparts (both *P* < 0.0001). In the multivariable analysis, the likelihood of disease-free status increased substantially with the number of healthy factors present across different subgroups (*P* < 0.0001). That is, men displaying six to ten healthy factors were 2.05–8.76 times more likely to be classified as disease-free status compared to those with two or fewer, while the corresponding number for women was 1.63–3.54. Each additional healthy factor was associated with a 15–17% higher likelihood of disease-free status. Men with six or more healthy factors had 1.0–2.5 longer disease-free years compared with those with two or less. The corresponding number for women was 0.6–2.0 years (Figure 5).

### Sensitivity Analysis

Cross-sectional analysis of 152,813 participants showed that the leading predictors for disease-free were similar to those obtained in the longitudinal analysis, although in different orders. Overweight/obesity, physical inactivity, smoking, passive smoking, and diets low in vegetables and fruits and high in red meat and chicken were associated with a lower likelihood of disease-free (Table S5). Modifiable factors accounted for 30.0–40.0% of total variance as derived from Random Forest. Self-rated health and quality of life were the two leading predictors of disease-free across subgroups (Figure S1). The results for leading predictors from other methods can be seen in Table S6.

## Discussion

In the present study, we report that approximately half of all participants remained disease-free status over a mean 9-year follow-up. The six leading predictors for disease-free status in both men and women were BMI, self-rated health, self-rated quality of life, red meat intake, chicken intake, and age. Participants with healthy diet habits, high physical activity, non-smokers, moderate alcohol consumption, moderate sleep time, a high socioeconomic status, and low psychological distress had a higher likelihood of disease-free status. A greater number of modifiable healthy factors was associated with a higher likelihood of disease-free status and longer disease-free years, highlighting the importance of intervention on these factors.

Our study agrees with previous studies (24–27), showing that men had a higher incidence of cardiometabolic disorders, Parkinson's disease, osteoarthritis, and hip replacement, while women were more likely to develop depression and asthma. However, unlike some studies (28), we found women had a higher incidence of cancer than men. This may be partly explained by the age range of our study participants as women aged 45–54 years had a higher incidence of cancer than their male counterparts in Australia (29). In the rankings of leading predictors, age moved from the sixth position in men aged 45–54 years to first at 55–64 years and from sixth to third in women. We argue that men are more affected by chronological age than women, which is consistent with previous studies that women had an advantage in life expectancy than men (30). This gender difference might be partly attributed to the more healthy factors clustered in women than men.

We observed BMI was the leading risk factor for disease-free status. This is consistent with a recent multi-cohort study showing that obesity was associated with a loss of 1.0–2.5 in 10 potential disease-free years during middle and later adulthood (31). Whilst, having a high BMI, was ranked as the fourth leading contributor to the global burden of disease in 2015, accounting for 4.9% of disability-adjusted life years (32). The increasing prevalence of overweight/obesity in both children and adults during 1980–2015 indicates that overweight/obesity represents a health challenge in the long-term (33). The increasing trend in BMI might be curved by healthy diets or high physical activity, which may need to be intervened by governments (34). We found that diets high in fruits and vegetables and low in red meat may help promote disease-free status, which is consistent with previous studies investigating chronic disease and mortality (32, 35). We also report that chicken intake was inversely associated with disease-free status likelihood, being among the leading five predictors in both women and men. This finding might be explained by the large proportion of chicken being fried, which contains higher levels of trans-fats and energy density resulting in an increased risk of chronic disease (35, 36). Reduction of red meat and fried chicken consumption deserves scrutiny for public health strategies to promote disease-free status.

Although there has been a decreasing trend in smoking prevalence in Australia (35), levels of passive smoking (29.1%), particularly in public places (25.5%), were high in our study. We observed, on average, one current smoker affects three passive smokers and our multivariable analysis demonstrated current smoking accounted for 3.9% of the incidence of chronic conditions, compared with 1.8% caused by passive smoking. Thus, policy-responsive passive smoking control also deserves scrutiny, given both direct and passive smoking are major threats to disease-free status.

The likelihood of disease-free status increased substantially with an increased number of modifiable healthy factors in our study. We also observed a low proportion of participants with more than six healthy factors, suggesting public health interventions promoting modifiable healthy factors would likely help curb the increasing incidence of chronic conditions in the aging population. Our study also demonstrated that participants having six or more modifiable healthy factors had 0.60–2.49 more disease-free years out of a 9-year follow-up. This underlines that modifications on these healthy factors may help maximize disease-free status in middle-aged individuals. Participants with lower socioeconomic status are inevitably more likely to display higher rates of unhealthy behaviors, be of elevated psychological distress and less affordable and accessible to healthy foods or built environments in physical activity (34, 37). Improving modifiable healthy factors among these vulnerable people should be a priority.

Self-reported overall and health quality of life are stronger predictors for disease-free status than psychological distress or individual socioeconomic factors, including income, education, health insurance, and relative socioeconomic disadvantage in our study. This may be attributable to the fact that self-rated health, a measure of socioeconomic inequality, also reflects the perception of the biological and psychological status of individuals in given cultural and social circumstances (38). Individuals at different stages of life may differ in the evaluation of their health status (38). This is consistent with our findings that self-rated health and quality of life ranked lower as a predictor for disease-free status in individuals aged 55–64 years compared with those aged 45–54 years. Consistent with previous studies (39), the hazardous effects of psychological distress on disease-free status was observed in our study, particularly among the younger population.

To our knowledge, this is the first study to comprehensively examine associations of multiple predictors including biological, socioeconomic, psychological, and geographic factors with disease-free status in a community-dwelling population with large sample size and long-term follow-up. We exploited multiple machine learning methods to analyze the leading predictors given the low prediction performance of traditional regression model (18, 40) and examined the association of clustering modifiable healthy factors with disease-free status.

Some limitations should also be considered. Firstly, we did not utilize the traditional definition of disease-free status that involves physical conditions, and self-rated mental and cognitive function. Despite this, we included the majority of chronic conditions that contribute to mortality and caused by impairment of physical, mental, or cognitive function. Secondly, although some chronic conditions that may be related to worse healthy aging were not included in our analysis because of the unavailability, the involved conditions contributed to a predominant proportion of total mortality in Australia (6). Thirdly, all data regarding exposures (apart from geographic information) were self-reported; therefore, we cannot deny the potential influences of self-reporting bias. However, the measurement errors would be more likely to bias true associations to the null as the data were collected before any of the chronic conditions of interest occurred. Fourthly, participants in our study were, on average, healthier than the general population in New South Wales; however, similar associations between exposures and health outcomes in this cohort study have been reported previously compared with a population representative study (20). Fifthly, the definition of incident chronic conditions based on MBS and PBS data in our study may be biased because the awareness of diagnosis of a disease is dependent of health care seeking behavior and accessibility, although we controlled the related confounders including education, household income, health insurance, psychological distress, overall health, geographic remoteness, family history of chronic diseases, age, and gender in the multivariable-analysis. Sixthly, the definition of chronic conditions was based on both self-reported and MBS/PBS data at baseline but MBS/PBS data only during follow-up, which might have introduced some bias. Seventhly, it seems that disease events for individuals with general beneficiaries might be less likely to be captured using PBS data compared to those with concessional beneficiaries before July 2012 (41). However, the combination of PBS and MBS data to detect chronic conditions in our study might have largely reduced this bias, which is reflected in the gradual decrease trend of disease-free status without sharp decrease over 10 years in Figure 2. Eightly, although the participation rate of our study was similar to previous studies of this kind (42, 43), the relatively low participation rate (18%) might limit the generalization of our findings.

In conclusion, despite chronological age plays an important role in disease-free status, modifiable factors including BMI, diets, physical activity, direct, and indirect smoking, and alcohol consumption accounted for a predominant proportion of total variance suggesting that improvement in healthy behaviors may substantially promote disease-free status in the middle-age population. Participants with low socioeconomic status, high psychological distress, or poor/fair self-rated health are more in need of health services and social support. The findings provide evidence on priorities of health strategy to promote disease-free status in middle-aged men and women, resulting in increased population longevity in the long-term.

## Data Availability Statement

The datasets for this manuscript are not publicly available because The data that support the findings of this study are available from The Sax Institute but restrictions apply to the availability of these data, which were used under license for the current study, and so are not publicly available. Data are however available from the authors upon reasonable request and with permission of The Sax Institute. Requests to access the datasets should be directed to MH, mingguang.he@unimelb.edu.au.

## Ethics Statement

The 45 and Up study has ethical approval from the UNSW Human Research Ethics Committee. The study protocol was approved by the Royal Victorian Eye and Ear Hospital Human Research Ethics Committee. Participants provided consent to follow-up and link their data to routine health datasets.

## Author Contributions

XS, LZ, and MH conceived and designed the research. XS and LZ conducted data analysis and interpretation. XS wrote the initial draft of the manuscript. XS, LZ, WW, SK, JW, and MH revised the manuscript. All authors read and approved the final manuscript.

### Conflict of Interest

The authors declare that the research was conducted in the absence of any commercial or financial relationships that could be construed as a potential conflict of interest.
