# Supplementary material for: Leading Determinants for Disease-Free Status in Community-Dwelling Middle-Aged Men and Women: A 9-Year Follow-Up Cohort Study
Source: Front Public Health. 2019 Nov 8;7:320. doi: 10.3389/fpubh.2019.00320 (PMC6857185; doi:10.3389/fpubh.2019.00320)
Supplement: Supplementary file 1 [file Data_Sheet_1.docx]

1. **Supplementary methods**
2. **Figure S1. Age- and gender-specific 20 leading predictors for ‘disease-free’ status derived from random forest in 152,813 participants**
3. **Table S1 List for Pharmaceutical Benefits Scheme and Medicare Benefits Schedule codes**
4. **Table S2. Combinations of the hyper-parameters with best performance for machine learning methods**
5. **Table S3. Area under curve by different machine learning methods**
6. **Table S4. Twenty leading predictors by different machine learning methods in the longitudinal analysis**
7. **Table S5. Relative risks for disease-free in middle aged men and women in the cross-sectional analysis (n=152,813)**
8. **Table S6. Twenty leading predictors by different machine learning methods in the cross-sectional analysis (n=152,813)**

**Supplementary methods**

**Classification of Independent Variables**

This section describes how the baseline data for independent variables were collected and the classification of these variables.

Participants were classified as current, former and never smokers based on two questions: “Have you ever been a regular smoker?” and “Are you a regular smoker now?” Passive smoking was self-reported in hours per week at home or in other places and was divided in two groups as passive smoking or not.

Alcohol intake was calculated based on two questions “About how many alcoholic drinks do you have each week?” and “On how many days each week do you usually drink alcohol?” Responses were then categorized according to number of drinks per week: 0, 1-4, 5-7, 8-14, or >14 using the National Health and Medical Research Council definitions, with more than two drinks per day (14 drinks/week) as consuming “risky” levels of alcohol.

Physical activity was measured using the Active Australia Survey^1^ where the total time one spent on walking, moderate-intensity, and vigorous-intensity physical activity (bouts of at least 10 minutes) in the previous week was assessed. Questions were also asked about number of hours spent on sitting, watching television, sleep in a typical 24-hour day.

Sleep and sitting time was assessed using the question “About how many hours in each 24 hour day do you usually spend doing the following: sleeping, sitting and standing?” Sleep time was divided into three groups: <7, 7-9, and >9 hours. Sitting and standing time were categorized into two groups as ≤8 or >8 hours per day.

Outdoor per day was assessed using the question “About how many hours a day would you usually spend outdoors on a weekday and on the weekend?” An average outdoor time per day in one week in hours was calculated based on the time spent on workdays and weekend days and was categorized in five groups according to the quintiles.

Frequency of dietary intakes including vegetable, fruit, breakfast cereal, milk, fish, chicken, red meat, and processed meat per week or per day was recorded based on separate questions.

Socioeconomic status was also assessed using the Index of Relative Socio-economic Disadvantage according to postcode that ranks the income, qualifications, and skilled occupations of residents within an area.^2^ Participants were divided into five groups according to the quintiles of Index of Relative Socio-economic Disadvantage, with the lowest quintile representing the greatest socio-economic disadvantage.

Geographic remoteness was divided into four groups including major cities, inner regional area, outer regional area, and remoteness using the Accessibility Remoteness Index of Australia.^3^

Psychological distress was accessed using the Kessler-10 scale,^4^ which provides a global measure of anxiety and depressive symptoms experienced in the preceding month. Scores range from 10 to 50, with the following categories: low (10-11), mild (12-15), moderate (16-21) and high (22-50) psychological distress.

Self-reported quality of life was classified as excellent, very good, good, fair, or poor based on the question “In general, how would you rate your quality of life?” While, self-reported overall health was classified as excellent, very good, good, fair, or poor based on the question “In general, how would you rate your overall health?”

Four questions from the Duke Social Support Scale asked the respondent how many times per week they spend: time with friends or family they do not live with (0, 1-2, and ≥3 were scored as 1, 2, and 3, respectively); talk to someone (friends, relatives or others) on the telephone (0-1, 2-5, and ≥6 were scored as 1, 2, and 3, respectively); at meetings of social clubs, religious/other groups (0-1, 2-5 and ≥6 were scored as 1, 2 and 3, respectively); and how many people outside home, within 1 hour of travel they can depend on or feel very close to (0, 1-2 and ≥3 were scored as 1, 2 and 3 respectively) ^5^. The total social interaction score ranged from 4-12 and was categorized as low (4-6), moderate (7-9), and high levels (10-12).

Family history of chronic diseases including heart disease, stroke, hypertension, cancer, diabetes, Alzheimer’s, Parkinson’s disease, depression, arthritis, osteoporosis, and hip fractures were self-reported.

**Description of Machine Learning Methods**

This section describes how we applied four machine learning methods to evaluate the importance of predictors.

**Factorization of features**

The data were factorized into a labelled dataset containing the independent variables (potential predictors were listed in Table 1) and the dependent variable (participants were free of 13 chronic conditions in the 9 years following the baseline date) using h2o. We used the whole dataset as both training and testing data as we aimed only to obtain the variable importance metric. We applied four commonly used machine learning methods including logistic regression, random forest, gradient boosting machine and deep learning.

**Logistic regression**

Logistic regression model was used to analyze the importance of the predictors. The hyper-parameters alpha and lambda specify the regularization strength and the regularization distribution between L1 and L2 penalties respectively. A five-fold cross-validation was applied to test if the model was overfitting and a grid search to obtain optimal parameters including alpha and lambda was conducted.

**Random forest**

Random forest has been widely applied in research since its creation. The random forest algorithm is a supervised learning algorithm constructing an ensemble of decision-trees using randomly bootstrapping sample datasets and averaging predictions of its trees.^6^ It applies a bagging method to ensemble multiple decision trees generated from subsets to reduce correlations among the constitute decision trees. A lower correlation between decision tress is associated with a lower forest error rate. Random forest has its robustness to reduce noise and overfitting, given that the datasets are built independently using bagging method.^6,7^ The strength of each individual tree in the forest is another determinist factor for the forest error rate. In this study, we used the Area under curve to determine the best predicting variable and location for each tree split in our algorithm. We grew the forest with a 500 trees. A five-fold cross-validation was conducted to test if the model was overfitting even though random forest is less likely to be overfitting compared with other methods.^6^ We implemented grid search to obtain optimal parameters including the number of variables randomly sampled as candidates at each split and the max depth of each tree (effectively the number of interactions are considered in the model) for random forest. A range of values for each hyper-parameters were specified and all possible combinations of the hyper-parameters were examined and the combination with the highest cross-validation performance metric was obtained. There are several indices for the model performance and maximization of the area under the receiver operating characteristic curve was applied in this study. For example, random forest has hyper-parameters specifying the number of trees and the max depth of each tree (effectively how many interactions are considered in the model), whereas the decision rules are the parameters.

**Gradient boosting machine**

Gradient boosting machine belongs to a family of machine learning approaches leveraging a boosting ensemble method. An ensemble of decision-trees were constructed using a weighted average of trees with more weight to those with better performance.^8^ Gradient boosting machine converts a weak original learning algorithm to a strong one by minimizing an exponential loss of the misclassification rate. Compared with random forest with low probability of being overfitting, gradient boosting machine has its advantage in reducing bias.^9^ We applied 500 trees and fivefold cross-validation in the model. A grid search for model optimization was also conducted with the maximum number of models, the max depth of each tree, learning rate, row sample rate per tree, and column sample rate as hyper-parameters.

**Deep learning**

A multi-layer deep learning is also known as a deep neural network that exploits multiple layers of non-linear information processing for automatic feature extraction, pattern analysis, and classification.^10,11^ It applies algorithms for learning patterns by modeling hierarchical multiple layers of representation among high dimensional data derived from lower-level ones.^10^ There are many parameters that influence on the performance of the deep learning model. One parameter specifies the activation function, which helps the network exploit the useful information and suppress the irrelevant data points. Several types of activation function include "Tanh", "TanhWithDropout", "Rectifier", "RectifierWithDropout", "Maxout", and "MaxoutWithDropout". Another important parameter is the hidden layer, which would implement the processing and transform the final output to the output layer when the information moves from the input layer to the hidden layers. The parameter ‘epochs’ denotes how many times the dataset should be iterated. A constraint for squared sum of incoming weights per unit of 10 and a relative tolerance for metric-based stopping criterion of 1% were applied. A grid search and fivefold cross-validation was also conducted for deep learning with activation, hidden layer size, L1 and L2 regularization, and input dropout ratio as hyper-parameters. The following parameter specifies the activation function: "Tanh", "TanhWithDropout", "Rectifier", "RectifierWithDropout", "Maxout", and "MaxoutWithDropout". Both L1 regularization would add stability and improve generalization, whereas L1 causes many weights to become 0 and L2 causes many weights to be small.

We set the parameter nthreads as -1 as to make use of all available cores on the system.


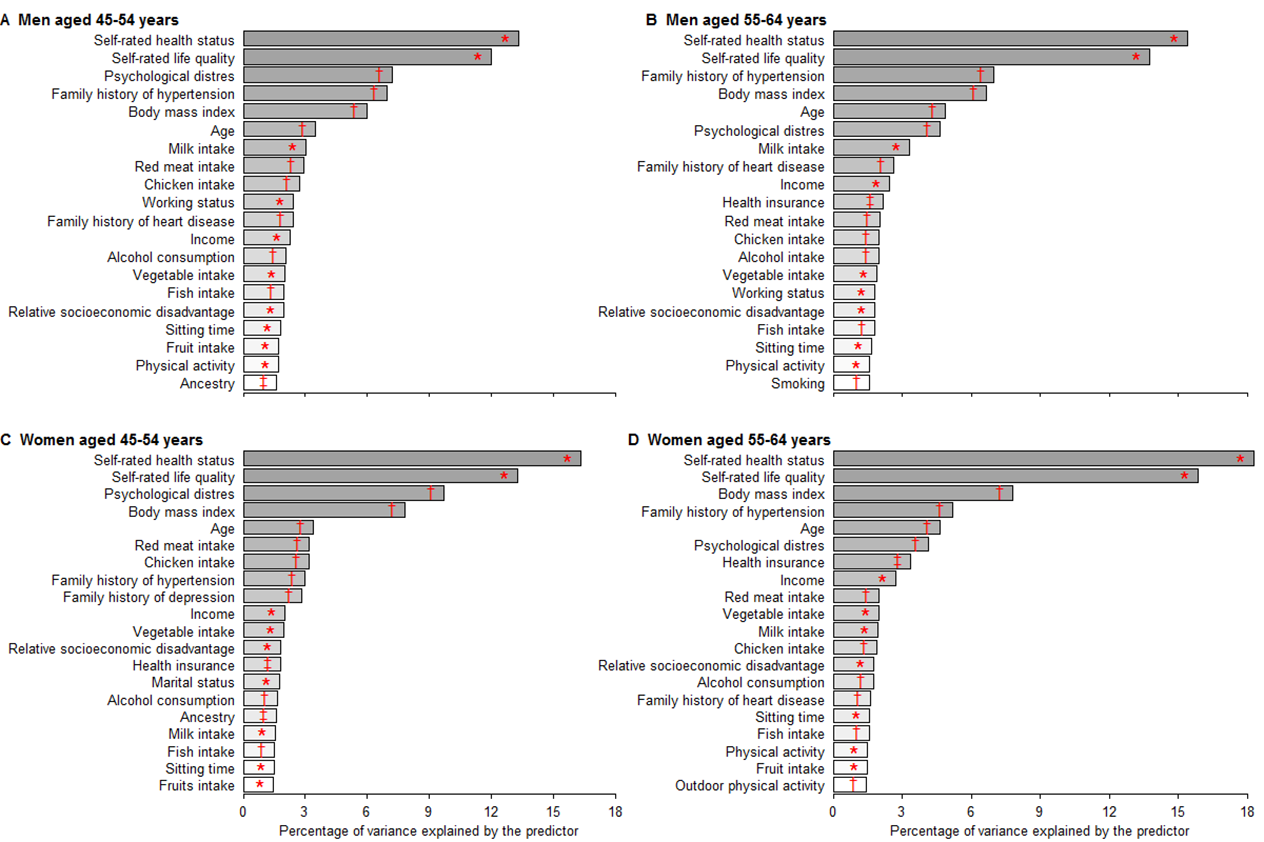


**Figure S1. Age- and Gender-specific 20 Leading Predictors for ‘Disease-free’ Status derived from Random Forest in 152,813 Participants**

‘Disease-free’ status was defined as participants without developing any of the thirteen chronic conditions at baseline. Panels A, B, C and D show the 20 leading predictors for disease-free in men aged 45-54 years and 55-64 years and women aged 45-54 years and 55-64 years. Machine learning methods including random forest, gradient boosting machine, deep learning and logistic regression were applied to evaluate the importance of predictors and results from random forest with the best prediction performance are shown in this figure.

^e^Variables were inversely associated with disease-free proportion.

^f^Variables were positively associated with disease-free proportion.

^g^Variables were nonlinearly associated with disease-free proportion.

**Table S1 List for Pharmaceutical Benefits Scheme and Medicare Benefits Schedule Codes**

| **Chronic conditions** | **Pharmaceutical Benefits Scheme codes** | **ATC codes for Pharmaceutical Benefits Scheme** | **Medicare Benefits Schedule codes** | **Corresponding ICD codes** |
| --- | --- | --- | --- | --- |
| Heart disease |  |  | 13818, 13842, 13847, 13848, 13857, 13400, 13830, 13847, 13851, 13854, 13839, 38497, 38498, 38499, 38500, 38501, 38502, 38503, 38504, 38200, 38203, 38206, 38209, 38212, 38213, 38215, 38218, 38220, 38222, 38225, 38228, 38231, 38234, 38237, 38240, 38241, 38243, 38246, 38256, 38270, 38272, 38273, 38274, 38275, 38285, 38286, 38287, 38290, 38293, 38300, 38303, 38306, 38309, 38312, 38315, 38318, 38470, 38473, 38496, 38497, 38498, 38500, 38501, 38503, 38504, 38505, 38506, 38507, 38508, 38509, 38512, 38515, 38518, 38588, 38600, 38603, 38609, 38612, 38613, 38615, 38618, 38621, 38624, 38627 | I20-I52 |
| Stroke |  |  | 32700, 32703, 32760, 33500, 33545, 33548, 33551, 33554, 35303, 35307, 39800, 39803, 39806, 39812, 39815, 39818, 39821, 18365, 18364, 35414, 33800, 34100 | I60-I64 |
| Hypertension^a^ | 1629R, 3145M, 3141H, 9019Q, 9020R, 1478T, 1480X, 1479W, 1639G, 1640H, 2313R, 1484D, 1585K, 8532C, 2436F, 1486F, 1280J, 2752W, 2751T, 2366M, 2367N, 2361G, 8679T, 8534E, 8610E, 1907J, 1695F, 1694E, 1906H, 1241H, 2208F, 1248Q, 1250T, 1313D, 8480H, 1312C, 1335G, 1149L, 1147J, 1148K, 8760C, 1370D, 1368B, 1369C, 1183G, 1182F, 2458J, 2456G, 2457H, 9006B, 3050M, 3051N, 9007C, 8704D, 9008D, 1969P, 1968N, 1970Q, 8470T, 1316G, 9120B, 1944H, 1945J, 9121C, 1946K, 9122D, 8758Y, 2792Y, 2791X, 2793B, 8477E, 8401E, 2190G, 2845R, 8449Q, 8590D, 8589C, 9145H, 9144G, 9347Y, 9349C, 9348B, 9346X, 2626F, 2629J, 2857J, 9387C, 8297Q, 8295N, 8889W, 8296P, 8447N, 8397Y, 5491B, 8951D, 8247C, 8248D, 8246B, 5452Y, 8203R, 2148C, 2147B, 8355R, 8356T, 9369D, 9370E, 9368C, 9371F, 9314F, 8504N, 9315G, 8624X, 8405J, 8404H, 2136K, 2170F, 2166B, 2161R, 8622T, 8623W, 9381R, 9372G, 9373H, 9374J, 9482C, 9481B, 9377M, 9375K, 5459H, 9376L, 5460J, 5294P, 5292M, 5293N, 8979N, 8980P, 8978M, 8981Q, 5289J, 5286F, 5287G, 5285E, 5288H, 2953K, 10005N, 2864R, 2880N, 2836G, 9056P, 9054M, 9050H, 9051J, 9052K, 9053L, 9049G, 9055N | C02AB01, C02AC01, C02AC05, C02CA01, C02DB02, C02DC01, C03AA03, C03BA04, C03BA11, C03EA01, C08CA01, C08CA02, C08CA05, C08CA13, C08DA01, C08DB01, C09AA01, C09AA02, C09AA03, C09AA04, C09AA05, C09AA06, C09AA09, C09AA10, C09BA02, C09BA04, C09BA06, C09BA09, C09BB02, C09BB04, C09BB05, C09BB10, C09CA01, C09CA02, C09CA03, C09CA04, C09CA06, C09CA07, C09CA08, C09DA02, C09DA03, C09DA04, C09DA06, C09DA07, C09DA08, C09DB01, C09DB02, C09DB04, C09DX01, C09DX03 |  | I10, I11-I13, I15 |
| Dyslipidemia^a^ | 8215J, 8213G, 8521L, 8214H, 9232X, 9230T, 9233Y, 9231W, 2863Q, 9236D, 2834E, 2833D, 8197K, 8829Q, 9238F, 9237E, 9239G, 9240H, 2574L, 2628H, 2606E, 2594M, 9044B, 9043Y, 9042X, 9045C, 2609H, 2584B, 2590H, 2636R, 3404E, 3403D, 3402C, 3405F, 8313M, 2011W, 2013Y, 8173E, 2012X, 9245N, 9242K, 9241J, 9244M, 9243L, 9022W, 9023X, 9246P, 9247Q, 1453L, 9248R, 2967E, 9249T, 10958R, 8757X, 10393B, 10376D, 10377E, 10392Y, 8882L, 8881K, 9484E, 9483D, 10207F, 10208G, 10201X, 10204C, 9056P, 9054M, 9050H, 9051J, 9052K, 9053L, 9049G, 9055N | C10AA01, C10AA03, C10AA04, C10AA05, C10AA07, C10AB04, C10AB05, C10AC01, C10AX09, C10AX13, C10BA02, C10BA05, C10BA06, C10BX03 |  | E78 |
| Diabetes^a^ | 8571D, 8435Y, 9224L, 1921D, 8212F, 8084L, 1713E, 1531N, 1762R, 1711C, 1761Q, 1533Q, 8609D, 1426C, 1763T, 2062M, 8874C, 8390N, 9040T, 9039R, 2430X, 1801T, 3439B, 9435N, 8607B, 2939Q, 2449X, 9302N, 8535F, 8450R, 8451T, 8452W, 8533D, 2440K, 10033C, 10032B, 10035E, 10515K, 10510E, 10516L, 10649L, 10650M, 10640B, 10627H, 10626G, 10633P, 10677Y, 10038H, 10044P, 10045Q, 8838E, 8810Q, 8811R, 9061X, 9062Y, 9059T, 9060W, 10051B, 10055F, 10048W, 9450J, 10090C, 9451K, 9449H, 10089B, 5475E, 5476F, 5474D, 8189B, 8188Y, 8694N, 8696Q, 8695P, 2944Y, 2933J, 2986E, 3387G, 10128C, 8983T, 9182G, 9181F, 9180E, 3415R, 10888C, 10202Y, 10206E, 10011X, 3423E, 3424F, 3415R, 9224L | A10AB01, A10AB02, A10AB04, A10AB05, A10AB06, A10AC01, A10AC02, A10AD, A10AD01, A10AD04, A10AE04, A10AE05, A10BA02, A10BB01, A10BB07 A10BB09, A10BB12, A10BD02, A10BD03, A10BD07, A10BD08, A10BD10, A10BD11, A10BD13, A10BD15, A10BD20, A10BF01, A10BG03, A10BH01, A10BH02, A10BH03, A10BH04, A10BH05, A10BJ01, A10BK01, A10BK03 |  | E10-E14 |
| Cancer^b^ | 1031G, 1079T, 1080W, 1134Q, 1144F, 1145G, 1160C, 1161D, 1162E, 1164G, 1265N, 1336H, 1340M, 1342P, 1390E, 1811H, 1929M, 1930N, 1931P, 1932Q, 2198Q, 2199R, 2315W, 2371T, 2372W, 2374Y, 2381H, 2521Q, 2528C, 2548D, 2561T, 2578Q, 2579R, 2580T, 2581W, 2582X, 2583Y, 2585C, 2884T, 2885W, 2904W, 2910E, 3017T, 3026G, 4222F, 4223G, 4309T, 4319H, 4326Q, 4327R, 4357H, 4360L, 4361M, 4364Q, 4394G, 4402Q, 4403R, 4428C, 4429D, 4431F, 4433H, 4439P, 4448D, 4451G, 4502Y, 4512L, 4514N, 4531L, 4567J, 4600D, 4610P, 4613T, 4614W, 4615X, 4618C, 4619D, 4620E, 4632T, 4639E, 4650R, 4703M, 4706Q, 4712B, 4713C, 4725Q, 4732C, 5149B, 5156J, 5270J, 5271K, 5272L, 5273M, 5274N, 5275P, 5428Q, 5429R, 5430T, 5431W, 5432X, 5433Y, 5462L, 5463M, 5464N, 5485Q, 5486R, 5487T, 5488W, 5489X, 5581R, 5582T, 5583W, 5584X, 5585Y, 5586B, 5587C, 5588D, 5589E, 5590F, 5591G, 5592H, 5593J, 5594K, 5595L, 5596M, 5597N, 5598P, 5705G, 5801H, 5804L, 5807P, 5808Q, 5809R, 5810T, 5811W, 5812X, 5813Y, 5814B, 5833B, 5834C, 5835D, 5842L, 5843M, 5844N, 5845P, 5846Q, 5847R, 5852B, 5854D, 5855E, 5856F, 5859J, 5860K, 5861L, 5862M, 5864P, 5865Q, 5866R, 5867T, 5868W, 5869X, 5872C, 5873D, 5874E, 5875F, 5876G, 5879K, 5880L, 5881M, 5882N, 5883P, 5887W, 5889Y, 5891C, 5892D, 5896H, 5897J, 5903Q, 5906W, 5907X, 5908Y, 5909B, 5910C, 5911D, 5912E, 5914G, 5915H, 5916J, 5917K, 5918L, 5919M, 5920N, 5921P, 5922Q, 5925W, 5926X, 5927Y, 5931E, 5932F, 5933G, 5934H, 5935J, 5936K, 5937L, 5943T, 5944W, 5957M, 5958N, 5959P, 5962T, 5963W, 5964X, 5965Y, 5966B, 5973J, 5974K, 5975L, 5976M, 5977N, 5978P, 5979Q, 5980R, 5981T, 5982W, 5983X, 5988E, 5989F, 5990G, 5991H, 5992J, 5993K, 6007E, 6008F, 6009G, 6010H, 6249X, 6440Y, 6441B, 6444E, 6445F, 6446G, 6447H, 6497Y, 6687Y, 6688B, 6689C, 6690D, 6691E, 6692F, 6693G, 6694H, 6695J, 6696K, 6697L, 6698M, 6699N, 6700P, 6701Q, 6702R, 6703T, 6704W, 6705X, 6706Y, 6707B, 6708C, 6709D, 6710E, 6711F, 6713H, 6714J, 6716L, 6843E, 6844F, 6845G, 6846H, 6847J, 6848K, 6891Q, 6892R, 6893T, 6894W, 6895X, 6896Y, 7050C, 7051D, 7052E, 7053F, 7054G, 7055H, 7086Y, 7087B, 7088C, 7089D, 7222D, 7224F, 7225G, 7226H, 7227J, 7228K, 7229L, 7230M, 7234R, 7235T, 7237X, 7238Y, 7239B, 7244G, 7246J, 7248L, 7249M, 7250N, 7251P, 7252Q, 7254T, 7255W, 7256X, 7257Y, 7258B, 7259C, 7261E, 7262F, 7263G, 7264H, 7265J, 7266K, 7267L, 7268M, 7269N, 7270P, 7271Q, 7272R, 7274W, 7275X, 7281F, 7282G, 7283H, 7284J, 7285K, 8018B, 8033T, 8034W, 8049P, 8050Q, 8071T, 8074Y, 8076C, 8077D, 8120J, 8280T, 8281W, 8284B, 8293L, 8294M, 8360B, 8414W, 8415X, 8515E, 8569B, 8570C, 8665C, 8666D, 8800E, 8809P, 8827N, 8828P, 8850T, 8851W, 8852X, 8863L, 8967Y, 8986Y, 8987B, 8988C, 8989D, 8990E, 8991F, 8992G, 8995K, 8996L, 9005Y, 9117W, 9118X, 9119Y, 9130M, 9131N, 9282M, 9283N, 9284P, 9291B, 9341P, 9401T, 9402W, 9410G, 9414L, 9415M, 9463C, 9689Y, 9690B, 9691C, 9713F, 9729C, 10148D, 10150F, 10158P, 10165B, 10179R, 10193L, 10269L, 10270M, 10296X, 10324J, 10346M, 10362J, 10381J, 10383L, 10391X, 10401K, 10402L, 10423N, 10575N, 10576P, 10581X, 10583B, 10588G, 10589H, 10591K, 10593M, 10595P, 10597R, 10708N, 10710Q, 10720F, 10741H, 10743K, 10744L, 10811B, 10817H, 10829Y | L01AA01, L01AA02, L01AA03, L01AA06, L01AB01, L01AX03, L01BA01, L01BA03, L01BA04, L01BB02, L01BB03, L01BB04, L01BC01, L01BC02, L01BC05, L01BC06, L01CA01, L01CA02, L01CA04, L01CB01, L01CD01, L01CD02, L01DB01, L01DB07, L01DC01, L01XA01, L01XA02, L01XC02, L01XC03, L01XE01, L01XE06, L01XE07, L01XX05, L01XX19, L01XX32 | 32036, 32099, 32102, 32103, 32104, 32106, 32108, 30299, 30300, 30301, 30302, 30303, 42801, 42802, 42803, 42805, 42807, 42809, 31340, 52036, 52039, 52048, 52045, 52042, 31372, 31373, 31374, 31375, 31376, 37227, 35720, 13915, 13918, 13921, 13924, 13927, 13930, 13933, 13936, 13939, 13942, 13945, 13948, 15000, 15003, 15006, 15009, 15012, 15100, 15103, 15106, 15109, 15112, 15115, 15211, 15214, 15215, 15218, 15221, 15224, 15227, 15230, 15233, 15236, 15239, 15242, 15245, 15248, 15251, 15254, 15257, 15260, 15263, 15266, 15269, 15272, 15275, 15303, 15304, 15307, 15308, 15311, 15312, 15315, 15316, 15319, 15320, 15323, 15324, 15327, 15328, 15331, 15332, 15335, 15336, 15339, 15342, 15345, 15348, 15351, 15354, 15357, 15600, 15700, 15705, 15710, 15715, 15900, 16003, 16006, 16009, 16012, 16015, 16018 | C00-C97 (excluding C44) |
| Depression^a^ | 2418G, 2429W, 2417F, 1561E, 1358L, 1357K, 1012G, 1011F, 1013H, 2420J, 2421K, 2523T, 2522R, 8702B, 8703C, 8220P, 8700X, 8701Y, 10181W, 8270G, 1434L, 8174F, 8512B, 2242B, 2237R, 2236Q, 8837D, 8836C, 2856H, 2444P, 8003F, 1900B, 10234P, 10241B, 9366Y, 10231L, 10245F, 9367B, 9156X, 9155W, 8290H, 3059B, 1628Q, 1627P, 8513C, 8856D, 8883M, 8855C, 9365X, 8857E, 8583R, 8868R, 8302Y, 8301X | N06AA02, N06AA04, N06AA09, N06AA10, N06AA12, N06AA16, N06AB03, N06AB04, N06AB05, N06AB06, N06AB08, N06AB10, N06AB03, N06AF03, N06AF04, N06AG02, N06AX03, N06AX11, N06AX16, N06AX18, N06AX21, N06AX23 |  | F32, F33 |
| Anxiety^a^ | 3135B, 3134Y, 5355W, 5356X, 5372R, 5371Q, 4144D, 4145E, 3135B, 3134Y, 9432K, 9433L, 10181W, 5357Y, 5358B, 5373T, 5374W, 4150K , 4151L, 4216X, 4522B, 8700X, 8701Y, 8849R | N05BA01, N05BA04, N05BA08, N05BA12, N05BE01 |  | F41.1 |
| Parkinson's disease^a^ | 1110K, 1109J, 2544X, 10013B, 10027R, 3038X, 5031T, 2362H, 2227F, 2225D, 2228G, 2229H, 8219N, 2231K, 8218M, 2226E, 1245M, 1242J, 1255C, 8970D, 9345W, 8799D, 8797B, 9292C, 9344T, 8798C, 3016R, 1443Y, 8394T, 8393R, 9152Q, 9151P, 9153R, 3420B, 5143Q, 3418X, 5145T, 3421C, 3419Y, 3422D, 9394K, 9393J, 2410W, 2384L, 11140H, 2385M, 1952R, 1973W, 8367J, 9744W, 11083H, 9607P, 10235Q, 9640J, 10971K | N04AA01, N04AA02, N04AC01, N04BA02, N04BA03, N04BB01, N04BC05, N04BC06, N04BC07, N04BC09, N04BD01, N04BD02, N04BX02 | 40850, 40851, 40852, 40854, 40856, 40858, 40860, 40862, 40801, 40850, 40851, 40852, 40854, 40856, 40858, 40860, 40862 | G20, F02.3 |
| Dementia^a^ | 2532G, 2479L, 8495D, 8496E, 2463P, 2531F, 2537M, 8770N, 8772Q, 8771P, 2477J, 10538P, 2475G, 2526Y, 2551G, 2493F, 2494G, 9161E, 10541T, 8497F, 8500J, 9162F, 8498G, 8499H, 2513G, 2492E, 9306T, 1956Y | N06DA02, N06DA02, N06DA03, N06DA04, N06DX01 |  | F03.90 |
| Asthma^a^ | 8240Q, 8136F, 8239P, 8141L, 8796Y, 8625Y, 10015D, 10024N, 8750M, 10018G, 10008R, 2827T, 10007Q, 8430Q, 8518H, 8431R, 8517G, 8432T, 8432T, 8519J, 11129R, 11124L, 8409N, 8408M, 2070Y, 2071B, 2072C, 2065Q, 2066R, 1542E, 8238N, 8627C, 8628D | R03AC12, R03AC13, R03AK06, R03AK07, R03AK10, R03AK11, R03BA01, R03BA02, R03BB01, R03DC03 | 2546, 2547, 2552, 2553, 2558, 2559, 2664, 2666, 2668, 2673, 2675, 2677 | J45, J46 |
| Hip replacement |  |  | 47492, 47495, 47498, 47540, 49336, 49318, 49319, 49321, 49324, 49327, 49330, 49333, 49339, 49342, 49345, 49346 | 0SRB049 |
| Osteoarthritis^a^ | 1300K, 1299J, 5077F, 5076E, 2757D, 5128X, 2454E, 5126T, 1896T, 5202T, 1898X, 5204X, 1895R, 5201R, 1897W, 5203W, 3192B, 5124Q, 3190X, 5123P, 1590Q, 5136H, 1615B, 1674D, 1614Y, 1659H, 1658G, 5179N, 5176K, 5178M, 5177L, 1795L, 5186Y | M01AB01, M01AB05, M01AC01, M01AC06, M01AE01, M01AE02, M01AE03, M01AH01 |  | M15-M19 |

Abbreviations: ATC, Anatomical Therapeutic Chemical; ICD, International Classification of Diseases.

^a^Pharmaceutical Benefits Scheme codes for these conditions were consistent with the corresponding ATC codes listed in a previous publication based on the 45 and up study.^12^ We used PBS codes instead of ATC codes for diagnosis detection where each ATC code may include numerous PBS codes. Different PBS codes within one ATC code represent different doses, forms (pill or liquid), intake methods (oral intake or injection), and specific conditions, which helps distinguish the claim purposes for different conditions.

^b^Non-melanoma skin cancer was excluded in our analysis.

**Table S2. Combinations of the Hyper-parameters with Best Performance for Machine Learning Methods^a^**

|  | **Logistic regression** | **Random forest** | **GBM** | **Deep learning** |
| --- | --- | --- | --- | --- |
| Male 45-54 years | nfolds=5, alpha=0.5, lambda=0.01 | max_depth=9, mtries=3, seed = 1, nfolds=5, ntree = 500 | ntrees = 500, nfolds = 5, seed = 1, learn_rate=0.06, max_depth=2, sample_rate=0.7, col_sample_rate=0.6 | activation="MaxoutWithDropout", adaptive_rate = F, input_dropout_ratio=0.0, l1=4.8E-5, l2=5.7E-5, hidden=c(25, 25, 25, 25), epochs=500, nfolds=5, fold_assignment="Modulo" |
| Male 55-64 years | nfolds=5, alpha=0.2, lambda=0.01 | max_depth=7, mtries=4, seed = 1, nfolds=5, ntree = 500 | ntrees = 500, nfolds = 5, seed = 1, learn_rate=0.01, max_depth=6, sample_rate=0.6, col_sample_rate=0.1 | activation="MaxoutWithDropout", adaptive_rate = F, input_dropout_ratio=0.0, l1=4.8E-5, l2=5.7E-5, hidden=c(25, 25, 25, 25), epochs=500, nfolds=5, fold_assignment="Modulo" |
| Female 45-54 years | nfolds=5, alpha=0.6, lambda=1.0E-4 | max_depth=10, mtries=4, seed = 1, nfolds=5, ntree = 500 | ntrees = 500, nfolds = 5, seed = 1, learn_rate=0.07, max_depth=2, sample_rate=0.6, col_sample_rate=0.1 | activation="MaxoutWithDropout", adaptive_rate = F, input_dropout_ratio=0.0, l1=4.8E-5, l2=5.7E-5, hidden=c(25, 25, 25, 25), epochs=500, nfolds=5, fold_assignment="Modulo" |
| Female 55-64 years | nfolds=5, alpha=0.2, lambda=0.01 | max_depth=10, mtries=3, seed = 1, nfolds=5, ntree = 500 | ntrees = 500, nfolds = 5, seed = 1, learn_rate=0.07, max_depth=2, sample_rate=0.6, col_sample_rate=0.1 | activation="MaxoutWithDropout", adaptive_rate = F, input_dropout_ratio=0.0, l1=4.8E-5, l2=5.7E-5, hidden=c(25, 25, 25, 25), epochs=500, nfolds=5, fold_assignment="Modulo" |

^a^These combinations of the hyper-parameters with best performance would then be separately applied in the machine methods in the final analysis.

**Table S3. Area Under Curve by Different Machine Learning Methods^a^**

| **Variables** | **Random forest** | **Gradient boosting machine** | **Deep learning** | **Logistic regression** |
| --- | --- | --- | --- | --- |
| **Longitudinal analysis** | |  |  |  |
| Male aged 45-54 years | 0.809 | 0.673 | 0.635 | 0.619 |
| Male aged 55-64 years | 0.744 | 0.772 | 0.641 | 0.626 |
| Female aged 45-54 years | 0.821 | 0.657 | 0.632 | 0.619 |
| Female aged 55-64 years | 0.878 | 0.672 | 0.645 | 0.623 |
| **Cross-sectional analysis** | |  |  |  |
| Male aged 45-54 years | 0.827 | 0.703 | 0.688 | 0.677 |
| Male aged 55-64 years | 0.818 | 0.731 | 0.707 | 0.700 |
| Female aged 45-54 years | 0.804 | 0.729 | 0.698 | 0.687 |
| Female aged 55-64 years | 0.815 | 0.751 | 0.725 | 0.708 |

^a^The whole data were treated as training data to evaluate the importance of predictors and testing data to evaluate the area under curve for both cross-sectional and longitudinal analysis.

**Table S4. Twenty Leading Predictors by Different Machine Learning Methods in the Longitudinal Analysis^a^**

|  | Men aged 45-54 years | |  | Men aged 45-54 years | |  | Women aged 45-54 years | |  | Women aged 45-54 years | |
| --- | --- | --- | --- | --- | --- | --- | --- | --- | --- | --- | --- |
| Ranking | Predictor | Percentage of variance explained by |  | Predictor | Percentage of variance explained by |  | Predictor | Percentage of variance explained by |  | Predictor | Percentage of variance explained by |
| Gradient boosting machine | | |  |  |  |  |  |  |  |  |  |
| 1 | Body mass index | 20.0 |  | Age | 8.5 |  | Body mass index | 16.6 |  | Body mass index | 13.8 |
| 2 | Red meat intakes | 8.0 |  | Body mass index | 7.1 |  | Self-rated quality of life | 9.6 |  | Age | 9.5 |
| 3 | Self-rated quality of life | 7.3 |  | Chicken intakes | 4.8 |  | Self-rated health | 8.7 |  | Chicken intakes | 8.9 |
| 4 | Self-rated health | 6.6 |  | Red meat intakes | 4.5 |  | Chicken intakes | 7.3 |  | Self-rated health | 6.8 |
| 5 | Age^b^ | 5.6 |  | Self-rated health | 4.2 |  | Education | 6.9 |  | Self-rated quality of life | 6.6 |
| 6 | Smoking | 5.4 |  | Smoking | 4.1 |  | Smoking | 6.1 |  | Education | 5.9 |
| 7 | Chicken intakes | 4.5 |  | Self-rated quality of life | 4.1 |  | Age | 5.0 |  | Red meat intakes | 5.5 |
| 8 | Health Insurance | 3.8 |  | Health insurance | 3.4 |  | Red meat intakes | 4.1 |  | Health insurance | 3.7 |
| 9 | Psychological distress | 3.4 |  | Alcohol consumption | 3.0 |  | Psychological distress | 4.0 |  | Smoking | 3.4 |
| 10 | Alcohol consumption | 2.9 |  | Relative socioeconomic disadvantage | 2.9 |  | Family history of heart disease | 3.0 |  | Alcohol consumption | 2.9 |
| 11 | Family history of heart disease | 2.8 |  | Income | 2.9 |  | Sleep time | 2.4 |  | Milk intakes | 2.8 |
| 12 | Education | 2.2 |  | Fruit intakes | 2.9 |  | Health insurance | 2.2 |  | Family history of heart disease | 2.8 |
| 13 | Milk intakes | 2.2 |  | Vegetables intakes | 2.8 |  | Passive smoking | 1.9 |  | Income | 2.5 |
| 14 | Fruit intakes | 2.0 |  | Education | 2.7 |  | Alcohol consumption | 1.8 |  | Remoteness | 2.0 |
| 15 | Social interaction | 1.7 |  | Outdoor physical activity | 2.6 |  | Physical activity | 1.7 |  | Fruit intakes | 1.9 |
| 16 | Outdoor physical activity | 1.5 |  | Physical activity | 2.6 |  | Milk intakes | 1.6 |  | Social interaction | 1.8 |
| 17 | Vegetables intakes | 1.4 |  | Ancestry | 2.6 |  | Remoteness | 1.6 |  | Vegetables intakes | 1.7 |
| 18 | Income | 1.3 |  | Psychological distress | 2.5 |  | Fruit intakes | 1.5 |  | Ancestry | 1.5 |
| 19 | Relative socioeconomic disadvantage | 1.3 |  | Fish intakes | 2.5 |  | Relative socioeconomic disadvantage | 1.2 |  | Processed meat intakes | 1.4 |
| 20 | Sitting time | 1.2 |  | Family history of hypertension | 2.4 |  | Vegetables intakes | 1.1 |  | Physical activity | 1.2 |
| Deep learning | |  |  |  |  |  |  |  |  |  |  |
| 1 | Marital status | 5.9 |  | Social interaction | 6.2 |  | Alcohol consumption | 5.8 |  | Social interaction | 5.6 |
| 2 | Physical activity | 5.6 |  | Outdoor physical activity | 5.1 |  | Chicken intakes | 4.8 |  | Alcohol consumption | 5.2 |
| 3 | Alcohol consumption | 5.2 |  | Physical activity | 5.0 |  | Physical activity | 4.8 |  | Marital status | 4.3 |
| 4 | Number of children | 4.8 |  | Number of children | 5.0 |  | Marital status | 4.6 |  | Smoking | 4.3 |
| 5 | Chicken intakes | 4.6 |  | Self-rated quality of life | 4.9 |  | Residential rurality | 4.3 |  | Chicken intakes | 4.1 |
| 6 | Health Insurance | 4.3 |  | Alcohol consumption | 4.6 |  | Health Insurance | 4.2 |  | Self-rated quality of life | 3.8 |
| 7 | Social interaction | 4.0 |  | Body mass index | 4.0 |  | Outdoor physical activity | 4.2 |  | Physical activity | 3.8 |
| 8 | Working status | 3.5 |  | Chicken intakes | 3.9 |  | Social interaction | 3.8 |  | Outdoor physical activity | 3.5 |
| 9 | Outdoor physical activity | 3.3 |  | Health Insurance | 3.9 |  | Red meat intakes | 3.7 |  | Body mass index | 3.4 |
| 10 | Vegetables intakes | 3.1 |  | Residential rurality | 3.4 |  | Vegetables intakes | 3.7 |  | Number of children | 3.3 |
| 11 | Relative socioeconomic disadvantage | 3.1 |  | Marital status | 3.3 |  | Fish intakes | 3.7 |  | Health Insurance | 3.1 |
| 12 | Residential rurality | 3.0 |  | Red meat intakes | 3.2 |  | Number of children | 3.6 |  | Breakfast cereal intakes | 3.0 |
| 13 | Fish intakes | 3.0 |  | Age | 3.0 |  | Self-rated quality of life | 3.5 |  | Residential rurality | 3.0 |
| 14 | Breakfast cereal intakes | 2.9 |  | Relative socioeconomic disadvantage | 2.9 |  | Body mass index | 3.3 |  | Relative socioeconomic disadvantage | 2.9 |
| 15 | Sitting time | 2.9 |  | Breakfast cereal intakes | 2.5 |  | Passive smoking | 3.1 |  | Working status | 2.6 |
| 16 | Passive smoking | 2.8 |  | Smoking | 2.5 |  | Family history of diabetes | 3.1 |  | Self-rated health | 2.6 |
| 17 | Self-rated quality of life | 2.6 |  | Working status | 2.5 |  | Relative socioeconomic disadvantage | 3.0 |  | Vegetables intakes | 2.4 |
| 18 | Family history of depression | 2.6 |  | Family history of hypertension | 2.4 |  | Smoking | 2.6 |  | Family history of depression | 2.4 |
| 19 | Country of birth | 2.3 |  | Family history of diabetes | 2.4 |  | Psychological distress | 2.5 |  | Red meat intakes | 2.3 |
| 20 | Age | 2.3 |  | Fish intakes | 2.2 |  | Family history of depression | 2.3 |  | Income | 2.3 |
| Logistic regression | |  |  |  |  |  |  |  |  |  |  |
| 1 | Smoking | 11.8 |  | Age | 21.1 |  | Self-rated health | 18.4 |  | Age | 18.5 |
| 2 | Self-rated health | 11.8 |  | Smoking | 13.0 |  | Chicken intakes | 15.6 |  | Self-rated health | 14.3 |
| 3 | Chicken intakes | 10.3 |  | Chicken intakes | 11.9 |  | Smoking | 13.8 |  | Red meat intakes | 11.8 |
| 4 | Age | 10.2 |  | Milk intakes | 11.3 |  | Milk intakes | 13.4 |  | Body mass index | 11.0 |
| 5 | Body mass index | 9.7 |  | Family history of hypertension | 9.1 |  | Age | 12.1 |  | Chicken intakes | 10.5 |
| 6 | Red meat intakes | 8.9 |  | Body mass index | 8.2 |  | Body mass index | 10.4 |  | Milk intakes | 9.6 |
| 7 | Family history of heart disease | 7.5 |  | Self-rated quality of life | 8.0 |  | Family history of heart disease | 8.9 |  | Smoking | 9.6 |
| 8 | Health insurance | 7.3 |  | Self-rated health | 7.9 |  | Psychological distress | 8.6 |  | Family history of heart disease | 7.8 |
| 9 | Milk intakes | 7.1 |  | Red meat intakes | 7.8 |  | Education | 7.9 |  | Education | 7.4 |
| 10 | Psychological distress | 5.4 |  | Health insurance | 7.5 |  | Sleep time | 7.0 |  | Family history of stroke | 4.4 |
| 11 | Self-rated quality of life | 4.7 |  | Family history of heart disease | 7.1 |  | Red meat intakes | 6.2 |  | Health insurance | 4.2 |
| 12 | Family history of hypertension | 3.0 |  | Education | 6.6 |  | Health insurance | 5.7 |  | Social interaction | 3.9 |
| 13 | Relative socioeconomic disadvantage | 2.7 |  | Psychological distress | 4.9 |  | Family history of hip fracture | 4.5 |  | Income | 3.7 |
| 14 | Fruit intakes | 2.2 |  | Family history of stroke | 3.9 |  | Family history of stroke | 3.5 |  | Relative socioeconomic disadvantage | 3.3 |
| 15 | Education | 1.9 |  | Physical activity | 3.8 |  | Relative socioeconomic disadvantage | 3.5 |  | Psychological distress | 3.1 |
| 16 | Family history of diabetes | 1.4 |  | Passive smoking | 3.8 |  | Family history of hypertension | 3.5 |  | Family history of hypertension | 2.8 |
| 17 | Family history of depression | 1.4 |  | Family history of depression | 3.7 |  | Passive smoking | 3.3 |  | Alcohol consumption | 2.8 |
| 18 | Breakfast cereal intakes | 0.9 |  | Ancestry | 3.2 |  | Fruit intakes | 3.3 |  | Breakfast cereal intakes | 2.6 |
| 19 | Alcohol consumption | 0.6 |  | Income | 2.7 |  | Vegetables intakes | 3.1 |  | Ancestry | 2.3 |
| 20 | Family history of stroke | 0.6 |  | Vegetables intakes | 2.5 |  | Family history of Parkinson's disease | 2.5 |  | Remoteness | 1.9 |

^a^Machine learning methods were used to evaluate the importance of predictors according to gender and age.

^b^Age was treated as categorical variable with each 2 years as one group.

**Table S5. Relative Risks for Disease-free in Middle Aged Men and Women in the Cross-sectional Analysis (n=152,813)^a^**

|  | No. of disease-free  participants | No. of  participants | Proportion  of disease-free | Univariate analysis | |  | Multivariable analysis | |
| --- | --- | --- | --- | --- | --- | --- | --- | --- |
|  |  |  |  | Relative Risk (95% CI) | P value |  | Relative Risk  (95% CI) | P value |
| Age (per two years) |  |  |  | 0.86 (0.85-0.87) | <0.0001 |  | 0.86 (0.86-0.87) | <0.0001 |
| Country of birth |  |  |  |  | <0.0001 |  |  | <0.0001 |
| Australia | 38393 | 15928 | 33.1 | 1.00 |  |  | 1.00 |  |
| Others | 13409 | 36078 | 37.2 | 1.19 (1.17-1.22) |  |  | 1.17 (1.14-1.20) |  |
| *Income* |  |  |  |  | <0.0001 |  |  | <0.0001 |
| <20000 AUD | 3152 | 6122 | 19.6 | 1.00 |  |  | 1.00 |  |
| 20000-39999 AUD | 6707 | 2165 | 30.3 | 1.79 (1.70-1.87) |  |  | 1.28 (1.21-1.35) |  |
| 40000-69999 AUD | 11991 | 33625 | 35.7 | 2.28 (2.18-2.39) |  |  | 1.39 (1.32-1.46) |  |
| ≥70000 AUD | 20989 | 53293 | 39.4 | 2.67 (2.56-2.79) |  |  | 1.45 (1.38-1.54) |  |
| *Education level* |  |  |  |  | <0.0001 |  |  | <0.0001 |
| <10 years | 2890 | 2162 | 23.8 | 1.00 |  |  | 1.00 |  |
| High school/TAFE | 31716 | 95208 | 33.3 | 1.60 (1.53-1.67) |  |  | 1.15 (1.09-1.20) |  |
| University or higher | 16953 | 43936 | 38.6 | 2.02 (1.93-2.11) |  |  | 1.12 (1.07-1.18) |  |
| Insurance |  |  |  |  | <0.0001 |  |  | <0.0001 |
| Private with extras | 29140 | 81239 | 35.9 | 1.00 |  |  | 1.00 |  |
| Private no extras | 7485 | 9083 | 39.2 | 1.15 (1.12-1.19) |  |  | 1.14 (1.10-1.18) |  |
| Health care concession | 3523 | 1192 | 16.6 | 0.36 (0.34-0.37) |  |  | 0.54 (0.51-0.56) |  |
| None of the above | 11184 | 29241 | 38.3 | 1.11 (1.08-1.14) |  |  | 1.17 (1.14-1.21) |  |
| Residential rurality |  |  |  |  | <0.0001 |  |  | 0.0001 |
| Major cities | 27442 | 79580 | 34.5 | 1.00 |  |  | 1.00 |  |
| Inner regional | 17768 | 52745 | 33.7 | 0.97 (0.94-0.99) |  |  | 1.08 (1.05-1.11) |  |
| Outer regional | 5170 | 5737 | 32.9 | 0.93 (0.90-0.96) |  |  | 1.13 (1.08-1.17) |  |
| Remote | 518 | 572 | 33.0 | 0.93 (0.84-1.04) |  |  | 1.09 (0.97-1.22) |  |
| Relative socioeconomic disadvantage |  |  |  |  | <0.0001 |  |  | 0.0066 |
| 1st quintile | 7942 | 7757 | 28.6 | 1.00 |  |  | 1.00 |  |
| 2nd quintile | 9724 | 260 | 32.1 | 1.18 (1.14-1.22) |  |  | 1.04 (1.00-1.08) |  |
| 3rd quintile | 9778 | 8311 | 34.5 | 1.32 (1.27-1.36) |  |  | 1.07 (1.03-1.11) |  |
| 4th quintile | 9464 | 6321 | 36.0 | 1.40 (1.35-1.45) |  |  | 1.06 (1.02-1.10) |  |
| 5th quintile | 13622 | 35844 | 38.0 | 1.53 (1.48-1.58) |  |  | 1.03 (0.99-1.07) |  |
| Marital status |  |  |  |  | <0.0001 |  |  | <0.0001 |
| Married/Partner | 43213 | 21249 | 35.6 | 1.00 |  |  | 1.00 |  |
| Single/Widowed/Divorced | 8823 | 1564 | 28.0 | 0.70 (0.68-0.72) |  |  | 0.85 (0.82-0.87) |  |
| Working status |  |  |  |  | <0.0001 |  |  | <0.0001 |
| Working/retired | 42850 | 18679 | 36.1 | 1.00 |  |  | 1.00 |  |
| Home duty/unpaid work/unemployed/disabled or sick | 7423 | 8143 | 26.4 | 0.63 (0.62-0.65) |  |  | 0.88 (0.85-0.91) |  |
| Others | 1648 | 5543 | 29.7 | 0.75 (0.71-0.79) |  |  | 0.91 (0.85-0.97) |  |
| Number of children |  |  |  |  | 0.7546 |  |  | <0.0001 |
| None | 6531 | 9223 | 34.0 | 1.00 |  |  | 1.00 |  |
| One or more | 45218 | 32646 | 34.1 | 1.01 (0.97-1.04) |  |  | 1.08 (1.04-1.12) |  |
| Family history of cancer |  |  |  |  | <0.0001 |  |  | 0.0274 |
| No | 29250 | 84220 | 34.7 | 1.00 |  |  | 1.00 |  |
| Yes | 22786 | 68591 | 33.2 | 0.93 (0.92-0.96) |  |  | 0.97 (0.95-1.00) |  |
| Family history of diabetes |  |  |  |  | <0.0001 |  |  | <0.0001 |
| No | 40682 | 14471 | 35.5 | 1.00 |  |  | 1.00 |  |
| Yes | 11354 | 38340 | 29.6 | 0.76 (0.74-0.78) |  |  | 0.82 (0.80-0.84) |  |
| Family history of heart disease |  |  |  |  | <0.0001 |  |  | <0.0001 |
| No | 32342 | 84532 | 38.3 | 1.00 |  |  | 1.00 |  |
| Yes | 19694 | 68278 | 28.8 | 0.65 (0.64-0.67) |  |  | 0.71 (0.69-0.73) |  |
| Family history of hypertension |  |  |  |  | <0.0001 |  |  | <0.0001 |
| No | 27353 | 67909 | 40.3 | 1.00 |  |  | 1.00 |  |
| Yes | 24683 | 84901 | 29.1 | 0.61 (0.59-0.62) |  |  | 0.60 (0.59-0.61) |  |
| Family history of stroke |  |  |  |  | <0.0001 |  |  | <0.0001 |
| No | 40769 | 15135 | 35.4 | 1.00 |  |  | 1.00 |  |
| Yes | 11267 | 37675 | 29.9 | 0.78 (0.76-0.80) |  |  | 0.84 (0.82-0.86) |  |
| Body mass index |  |  |  |  | <0.0001 |  |  | <0.0001 |
| 15-18.4 kg/m2 | 606 | 497 | 40.5 | 0.88 (0.79-0.97) |  |  | 1.02 (0.92-1.14) |  |
| 18.5-24.9 kg/m2 | 22575 | 51718 | 43.7 | 1.00 |  |  | 1.00 |  |
| 25-29.9 kg/m2 | 18497 | 55675 | 33.2 | 0.64 (0.63-0.66) |  |  | 0.67 (0.65-0.69) |  |
| ≥30 kg/m2 | 7472 | 5049 | 21.3 | 0.35 (0.34-0.36) |  |  | 0.40 (0.39-0.42) |  |
| Smoking |  |  |  |  | <0.0001 |  |  | <0.0001 |
| Never | 32064 | 87765 | 36.5 | 1.00 |  |  | 1.00 |  |
| Former | 15644 | 51296 | 30.5 | 0.76 (0.74-0.78) |  |  | 0.82 (0.80-0.85) |  |
| Current | 4315 | 3703 | 31.5 | 0.80 (0.77-0.83) |  |  | 0.94 (0.90-0.98) |  |
| Passive smoking |  |  |  |  | <0.0001 |  |  | <0.0001 |
| Yes | 32897 | 92411 | 35.6 | 1.00 |  |  | 1.00 |  |
| No | 15141 | 48025 | 31.5 | 0.83 (0.81-0.85) |  |  | 0.91 (0.89-0.94) |  |
| Alcohol consumption |  |  |  |  | <0.0001 |  |  | <0.0001 |
| None | 13513 | 44360 | 30.5 | 1.00 |  |  | 1.00 |  |
| 1-4 sessions/week | 12171 | 33305 | 36.5 | 1.31 (1.28-1.35) |  |  | 1.15 (1.12-1.19) |  |
| 5-7 sessions/week | 8202 | 1649 | 37.9 | 1.39 (1.35-1.44) |  |  | 1.17 (1.13-1.21) |  |
| 7-14 sessions/week | 10312 | 28381 | 36.3 | 1.30 (1.26-1.34) |  |  | 1.11 (1.07-1.15) |  |
| ≥15 sessions/week | 7364 | 3443 | 31.4 | 1.05 (1.01-1.08) |  |  | 0.98 (0.95-1.02) |  |
| Physical activity |  |  |  |  | <0.0001 |  |  | <0.0001 |
| 0-4 sessions/week | 8107 | 6816 | 30.2 | 1.00 |  |  | 1.00 |  |
| 5-9 sessions/week | 14964 | 44556 | 33.6 | 1.17 (1.13-1.21) |  |  | 1.07 (1.04-1.11) |  |
| 10-14 sessions/week | 12348 | 35971 | 34.3 | 1.21 (1.17-1.25) |  |  | 1.07 (1.03-1.11) |  |
| ≥15 sessions/week | 15440 | 41608 | 37.1 | 1.36 (1.32-1.41) |  |  | 1.19 (1.15-1.23) |  |
| Outdoor physical activity |  |  |  |  | <0.0001 |  |  | <0.0001 |
| ≤12 hours/week | 13372 | 38720 | 34.5 | 1.00 |  |  | 1.00 |  |
| 12.9-20 hours/week | 14579 | 41858 | 34.8 | 1.01 (0.98-1.04) |  |  | 1.03 (1.00-1.06) |  |
| 20.1-30 hours/week | 10616 | 31714 | 33.5 | 0.95 (0.92-0.98) |  |  | 1.05 (1.02-1.09) |  |
| >30 hours/week | 12918 | 38508 | 33.6 | 0.96 (0.93-0.99) |  |  | 1.14 (1.10-1.17) |  |
| Sleep time |  |  |  |  | <0.0001 |  |  | <0.0001 |
| <7 hours | 7376 | 3565 | 31.3 | 1.00 |  |  | 1.00 |  |
| 7-9 hours | 42438 | 19724 | 35.5 | 1.21 (1.17-1.24) |  |  | 1.03 (1.00-1.07) |  |
| >9 hours | 1287 | 6538 | 19.7 | 0.54 (0.50-0.58) |  |  | 0.69 (0.64-0.74) |  |
| Sitting time |  |  |  |  | 0.0436 |  |  | 0.0070 |
| <8 hours | 35708 | 4730 | 34.1 | 1.00 |  |  | 1.00 |  |
| ≥8 hours | 13811 | 39848 | 34.7 | 1.03 (1.00-1.05) |  |  | 0.96 (0.94-0.99) |  |
| Breakfast cereal intakes |  |  |  |  | <0.0001 |  |  | 0.0098 |
| Non-high fiber | 6851 | 799 | 32.9 | 1.00 |  |  | 1.00 |  |
| High fiber | 34812 | 1390 | 34.3 | 1.06 (1.03-1.10) |  |  | 0.96 (0.92-0.99) |  |
| Milk intakes |  |  |  |  | <0.0001 |  |  | <0.0001 |
| None | 2567 | 7890 | 32.5 | 1.00 |  |  | 1.00 |  |
| Skimmed fat/reduced fat/soy milk | 30010 | 93684 | 32.0 | 0.98 (0.93-1.03) |  |  | 0.92 (0.87-0.97) |  |
| Whole milk | 18137 | 48262 | 37.6 | 1.25 (1.19-1.31) |  |  | 1.25 (1.18-1.32) |  |
| Chicken intakes |  |  |  |  | <0.0001 |  |  | <0.0001 |
| None | 2057 | 5789 | 35.5 | 1.00 |  |  | 1.00 |  |
| 1 serving per week | 8685 | 6509 | 32.8 | 0.88 (0.83-0.94) |  |  | 0.93 (0.87-0.99) |  |
| 2 servings per week | 14716 | 45454 | 32.4 | 0.87 (0.82-0.92) |  |  | 0.88 (0.83-0.94) |  |
| 3 or more servings per week | 15539 | 48832 | 31.8 | 0.85 (0.80-0.90) |  |  | 0.86 (0.81-0.92) |  |
| Fish intakes |  |  |  |  | <0.0001 |  |  | <0.0001 |
| None | 4448 | 2533 | 35.5 | 1.00 |  |  | 1.00 |  |
| 1 serving per week | 22039 | 62357 | 35.3 | 0.99 (0.95-1.03) |  |  | 0.96 (0.92-1.00) |  |
| 2 servings per week | 13144 | 40079 | 32.8 | 0.89 (0.85-0.93) |  |  | 0.87 (0.83-0.91) |  |
| 3 or more servings per week | 9596 | 191 | 31.8 | 0.85 (0.81-0.88) |  |  | 0.81 (0.77-0.85) |  |
| Red meat intakes |  |  |  |  | <0.0001 |  |  | 0.0188 |
| 0 or 1 serving per week | 6652 | 9600 | 33.9 | 1.00 |  |  | 1.00 |  |
| 2 servings per week | 8852 | 7193 | 32.6 | 0.94 (0.90-0.98) |  |  | 0.97 (0.93-1.01) |  |
| 3 or 4 servings per week | 17395 | 54056 | 32.2 | 0.92 (0.89-0.96) |  |  | 0.99 (0.96-1.03) |  |
| 5 or more servings per week | 8315 | 6548 | 31.3 | 0.89 (0.85-0.92) |  |  | 1.03 (0.99-1.08) |  |
| Vegetables intakes |  |  |  |  | <0.0001 |  |  | <0.0001 |
| 0 or 1 serving per day | 5374 | 6095 | 33.4 | 1.00 |  |  | 1.00 |  |
| 2 servings per day | 13933 | 39213 | 35.5 | 1.10 (1.06-1.14) |  |  | 1.00 (0.96-1.05) |  |
| 3 servings per day | 8650 | 4313 | 35.6 | 1.10 (1.06-1.15) |  |  | 0.98 (0.93-1.02) |  |
| 4 servings per day | 13186 | 39412 | 33.5 | 1.00 (0.96-1.04) |  |  | 0.92 (0.88-0.96) |  |
| 5 or more servings per day | 9519 | 414 | 31.3 | 0.91 (0.87-0.95) |  |  | 0.90 (0.86-0.94) |  |
| Fruits intakes ─ no. (%) |  |  |  |  | <0.0001 |  |  | 0.4714 |
| None | 3665 | 1605 | 31.6 | 1.00 |  |  | 1.00 |  |
| 1 serving per day | 16941 | 49751 | 34.1 | 1.12 (1.07-1.17) |  |  | 0.99 (0.94-1.04) |  |
| 2 servings per day | 16624 | 48838 | 34.0 | 1.12 (1.07-1.17) |  |  | 0.97 (0.92-1.02) |  |
| 3 or more servings per day | 12242 | 35315 | 34.7 | 1.15 (1.10-1.20) |  |  | 0.97 (0.93-1.03) |  |
| Social interaction |  |  |  |  | <0.0001 |  |  | 0.0024 |
| Low | 8664 | 5950 | 33.4 | 1.00 |  |  | 1.00 |  |
| Moderate | 30747 | 87728 | 35.1 | 1.08 (1.05-1.11) |  |  | 0.97 (0.94-1.00) |  |
| High | 9305 | 8488 | 32.7 | 0.97 (0.93-1.00) |  |  | 0.93 (0.90-0.97) |  |
| Psychological distress |  |  |  |  | <0.0001 |  |  | <0.0001 |
| Low | 23328 | 56574 | 41.2 | 1.00 |  |  | 1.00 |  |
| Mild | 19019 | 54756 | 34.7 | 0.76 (0.74-0.78) |  |  | 0.74 (0.72-0.76) |  |
| Moderate | 6401 | 4150 | 26.5 | 0.51 (0.50-0.53) |  |  | 0.51 (0.49-0.53) |  |
| High | 1608 | 828 | 14.9 | 0.25 (0.24-0.26) |  |  | 0.27 (0.26-0.29) |  |
| Self-rated health status |  |  |  |  | <0.0001 |  |  | <0.0001 |
| Excellent | 15058 | 28179 | 53.4 | 1.00 |  |  | 1.00 |  |
| Very good | 22529 | 59864 | 37.6 | 0.53 (0.51-0.54) |  |  | 0.60 (0.58-0.62) |  |
| Good | 11251 | 46347 | 24.3 | 0.28 (0.27-0.29) |  |  | 0.37 (0.35-0.38) |  |
| Fair | 1519 | 2769 | 11.9 | 0.12 (0.11-0.12) |  |  | 0.20 (0.18-0.21) |  |
| Poor | 85 | 7 | 5.6 | 0.05 (0.04-0.06) |  |  | 0.11 (0.09-0.14) |  |
| Self-rated quality of life |  |  |  |  | <0.0001 |  |  | <0.0001 |
| Excellent | 14824 | 27700 | 53.5 | 1.00 |  |  | 1.00 |  |
| Very good | 22223 | 58958 | 37.7 | 0.53 (0.51-0.54) |  |  | 0.60 (0.58-0.62) |  |
| Fair | 11051 | 45487 | 24.3 | 0.28 (0.27-0.29) |  |  | 0.36 (0.35-0.38) |  |
| Poor | 1496 | 2457 | 12.0 | 0.12 (0.11-0.13) |  |  | 0.20 (0.19-0.21) |  |
|  | 79 | 44 | 5.5 | 0.05 (0.04-0.06) |  |  | 0.11 (0.08-0.13) |  |

^a^Poisson regression model with robust variance was used to calculate relative risk (95% CI) and P values.

**Table S6. Twenty Leading Predictors by Different Machine Learning Methods in the Cross-sectional Analysis (n=152,813)^a^**

|  | **Men aged 45-54 years** | |  | **Men aged 45-54 years** | |  | **Women aged 45-54 years** | |  | **Women aged 45-54 years** | |
| --- | --- | --- | --- | --- | --- | --- | --- | --- | --- | --- | --- |
| **Ranking** | Predictor | Percentage of variance explained by | | Predictor | Percentage of variance explained by | | Predictor | Percentage of variance explained by | | Predictor | Percentage of variance explained by |
| Gradient boosting machine | |  |  |  |  |  |  |  |  |  |  |
| 1 | Self-rated health | 25.8 |  | Self-rated health | 29.9 |  | Self-rated health | 29.1 |  | Self-rated health | 34.5 |
| 2 | Psychological distress | 11.7 |  | Body mass index | 10.4 |  | Psychological distress | 15.1 |  | Self-rated quality of life | 12.7 |
| 3 | Family history of hypertension | 10.6 |  | Self-rated quality of life | 10.2 |  | Body mass index | 11.1 |  | Body mass index | 10.8 |
| 4 | Self-rated quality of life | 9.2 |  | Family history of hypertension | 9.5 |  | Self-rated quality of life | 7.5 |  | Family history of hypertension | 7.1 |
| 5 | Body mass index | 9.2 |  | Age | 7.7 |  | Age | 4.2 |  | Age | 6.3 |
| 6 | Age^b^ | 4.8 |  | Psychological distress | 6.6 |  | Family history of hypertension | 3.9 |  | Health insurance | 5.1 |
| 7 | Milk intakes | 4.3 |  | Milk intakes | 4.9 |  | Family history of depression | 3.6 |  | Psychological distress | 4.2 |
| 8 | Red meat intakes | 3.6 |  | Family history of heart disease | 3.2 |  | Red meat intakes | 3.4 |  | Milk intakes | 2.9 |
| 9 | Working status | 3.0 |  | Health insurance | 2.5 |  | Marital status | 3 |  | Income | 2.2 |
| 10 | Family history of heart disease | 3.0 |  | Red meat intakes | 1.6 |  | Chicken intakes | 2.4 |  | Red meat intakes | 2.2 |
| 11 | Family history of depression | 1.9 |  | Working status | 1.4 |  | Health insurance | 2.2 |  | Family history of heart disease | 2.2 |
| 12 | Chicken intakes | 1.5 |  | Smoking | 1.3 |  | Milk intakes | 2 |  | Family history of depression | 1.9 |
| 13 | Health insurance | 1.2 |  | Chicken intakes | 1.3 |  | Country of birth | 1.5 |  | Smoking | 0.9 |
| 14 | Fish intakes | 1.1 |  | Alcohol consumption | 1.2 |  | Family history of heart disease | 1.4 |  | Chicken intakes | 0.8 |
| 15 | Marital status | 1.0 |  | Income | 1.1 |  | Working status | 1.3 |  | Vegetables intakes | 0.8 |
| 16 | Alcohol consumption | 0.9 |  | Family history of depression | 0.9 |  | Vegetables intakes | 1.2 |  | Country of birth | 0.7 |
| 17 | Smoking | 0.8 |  | Fish intakes | 0.7 |  | Smoking | 1.1 |  | Alcohol consumption | 0.7 |
| 18 | Income | 0.8 |  | Remoteness | 0.6 |  | Income | 0.7 |  | Sleep time | 0.5 |
| 19 | Vegetables intakes | 0.6 |  | Family history of diabetes | 0.5 |  | Fish intakes | 0.7 |  | Ancestry | 0.3 |
| 20 | Family history of diabetes | 0.5 |  | Sleep time | 0.4 |  | Sleep time | 0.6 |  | Relative socioeconomic disadvantage | 0.3 |
| Deep learning | |  |  |  |  |  |  |  |  |  |  |
| 1 | Social interaction | 7.8 |  | Chicken intakes | 6.3 |  | Chicken intakes | 6.7 |  | Red meat intakes | 10.1 |
| 2 | Red meat intakes | 5.7 |  | Social interaction | 6.2 |  | Social interaction | 6.7 |  | Alcohol consumption | 6.2 |
| 3 | Passive smoking | 5.0 |  | Sitting time | 6.1 |  | Self-rated quality of life | 5.2 |  | Chicken intakes | 5.8 |
| 4 | Sitting time | 5.0 |  | Red meat intakes | 5.8 |  | Red meat intakes | 4.8 |  | Marital status | 5.4 |
| 5 | Chicken intakes | 4.6 |  | Passive smoking | 4.8 |  | Family history of cancer | 4.7 |  | Body mass index | 5.2 |
| 6 | Family history of cancer | 4.6 |  | Marital status | 4.4 |  | Working status | 4.3 |  | Fruits intakes | 5 |
| 7 | Self-rated quality of life | 4.2 |  | Alcohol consumption | 4.4 |  | Breakfast cereal intakes | 4.1 |  | Smoking | 4.7 |
| 8 | Working status | 3.8 |  | Working status | 4.3 |  | Alcohol consumption | 3.8 |  | Social interaction | 4.6 |
| 9 | Marital status | 3.6 |  | Family history of cancer | 4.1 |  | Marital status | 3.7 |  | Age | 4.6 |
| 10 | Fruits intakes | 3.5 |  | Family history of depression | 3.7 |  | Passive smoking | 3.4 |  | Passive smoking | 3.3 |
| 11 | Breakfast cereal intakes | 3.2 |  | Age | 3.6 |  | Vegetables intakes | 3.4 |  | Education | 2.9 |
| 12 | Alcohol consumption | 3.1 |  | Self-rated quality of life | 3.6 |  | Family history of depression | 3.4 |  | Fish intakes | 2.9 |
| 13 | Family history of depression | 2.9 |  | Vegetables intakes | 3.3 |  | Age | 3.3 |  | Vegetables intakes | 2.8 |
| 14 | Fish intakes | 2.8 |  | Fruits intakes | 2.9 |  | Relative socioeconomic disadvantage | 3.3 |  | Income | 2.6 |
| 15 | Milk intakes | 2.8 |  | Processed meat intakes | 2.7 |  | Sitting time | 3 |  | Physical activity | 2.5 |
| 16 | Age | 2.7 |  | Fish intakes | 2.6 |  | Body mass index | 2.5 |  | Number of children | 2.4 |
| 17 | Processed meat intakes | 2.6 |  | Education | 2.4 |  | Fish intakes | 2.5 |  | Family history of cancer | 2.4 |
| 18 | Family history of hypertension | 2.5 |  | Smoking | 2.1 |  | Fruits intakes | 2.5 |  | Family history of dementia | 2.2 |
| 19 | Vegetables intakes | 2.3 |  | Family history of hypertension | 2.1 |  | Family history of dementia | 2.4 |  | Self-rated health | 1.9 |
| 20 | Sleep time | 2.3 |  | Sleep time | 1.9 |  | Milk intakes | 2.2 |  | Family history of hypertension | 1.8 |
| Logistic regression | |  |  |  |  |  |  |  |  |  |  |
| 1 | Self-rated health | 10.3 |  | Self-rated health | 22.6 |  | Self-rated health | 19.3 |  | Self-rated health | 31.8 |
| 2 | Family history of hypertension | 5.3 |  | Milk intakes | 8.8 |  | Milk intakes | 7.6 |  | Milk intakes | 8.9 |
| 3 | Milk intakes | 4.5 |  | Family history of hypertension | 8.1 |  | Psychological distress | 4.2 |  | Age | 6.2 |
| 4 | Psychological distress | 4.0 |  | Age | 6.1 |  | Family history of depression | 2.7 |  | Family history of hypertension | 5.8 |
| 5 | Age | 2.2 |  | Psychological distress | 2.8 |  | Age | 2.5 |  | Body mass index | 2.5 |
| 6 | Family history of heart disease | 1.2 |  | Body mass index | 2.2 |  | Family history of hypertension | 2.4 |  | Family history of depression | 1.8 |
| 7 | Body mass index | 0.9 |  | Family history of heart disease | 2.1 |  | Marital status | 1.9 |  | Family history of heart disease | 1.3 |
| 8 | Family history of depression | 0.9 |  | Red meat intakes | 0.9 |  | Body mass index | 1.6 |  | Psychological distress | 1.2 |
| 9 | Red meat intakes | 0.7 |  | Working status | 0.8 |  | Red meat intakes | 1.3 |  | Red meat intakes | 1.2 |
| 10 | Working status | 0.6 |  | Family history of depression | 0.8 |  | Smoking | 0.9 |  | Smoking | 0.7 |
| 11 | Marital status | 0.5 |  | Family history of diabetes | 0.5 |  | Family history of heart disease | 0.8 |  | Fruits intakes | 0.6 |
| 12 | Chicken intakes | 0.3 |  | Alcohol consumption | 0.5 |  | Working status | 0.8 |  | Family history of diabetes | 0.3 |
| 13 | Family history of diabetes | 0.2 |  | Income | 0.4 |  | Chicken intakes | 0.4 |  | Income | 0.3 |
| 14 | Sleep time | 0.1 |  | Marital status | 0.3 |  | Country of birth | 0.4 |  | Education | 0.3 |
| 15 | Self-rated quality of life | 0.1 |  | Smoking | 0.3 |  | Family history of cancer | 0.2 |  | Relative socioeconomic disadvantage | 0.3 |
| 16 | Income | 0.1 |  | Self-rated quality of life | 0.2 |  | Relative socioeconomic disadvantage | 0.2 |  | Working status | 0.3 |
| 17 | Education | 0.0 |  | Education | 0.2 |  | Fruits intakes | 0.2 |  | Marital status | 0.2 |
| 18 | Smoking | 0.0 |  | Physical activity | 0.2 |  | Education | 0.2 |  | Physical activity | 0.1 |
| 19 | Alcohol consumption | 0.0 |  | Remoteness | 0.2 |  | Family history of diabetes | 0.1 |  | Health insurance | 0.1 |
| 20 | Relative socioeconomic disadvantage | 0.0 |  | Country of birth | 0.1 |  | Income | 0.1 |  | Breakfast cereal intakes | 0.1 |

^a^Machine learning methods were used to evaluate the importance of predictors according to gender and age.

^b^Age was treated as categorical variable with each 2 years as one group.

**References**

1. Australian Institute of Health and Welfare. The active australia survey a guide and manual for implementation analysis and reporting. Canberra2003.

2. Australian Bureau of Statistics. 2039.0 - Information paper: an introduction to socio-economic indexes for areas (SEIFA), 2006. Canberra2008.

3. Australian Population and Migration Research Centre. Accessibility/Remoteness Index of Australia. Adelaide: Australian Population and Migration Research Centre; 2012.

4. Kessler RC, Andrews G, Colpe LJ, et al. Short screening scales to monitor population prevalences and trends in non-specific psychological distress. Psychol Med 2002;32:959-76.

5. Goodger B, Byles J, Higganbotham N, Mishra G. Assessment of a short scale to measure social support among older people. Aust N Z J Public Health 1999;23:260-5.

6. Breiman L. Random forests. Machine Learning 2001;45(1):5–32.

7. Kuhn M, Johnson K. Applied predictive modeling. New York: Springer; 2013.

8. Y. Freund, R. E Schapire. A Decision-Theoretic Generalization of On-Line Learning and an Application to Boosting. J Comput Syst Sci 1997;55:119-39.

9. Natekin A, Knoll A. Gradient boosting machines, a tutorial. Front Neurorobot 2013;7:21.

10. Deng L. , Yu D. . Deep Learning: Methods and Applications: Foundations and Trends in Signal Processing; 2014.

11. Aramaki E, Miura Y, Tonoike M, et al. Extraction of adverse drug effects from clinical records. Stud Health Technol Inform 2010;160:739-43.

12. Lujic S, Simpson JM, Zwar N, Hosseinzadeh H, Jorm L. Multimorbidity in Australia: Comparing estimates derived using administrative data sources and survey data. PLoS One 2017;12:e0183817.
